# HIF-1α regulated pathomechanism of low birth weight through angiogenesis factors in placental *Plasmodium vivax *infection

**DOI:** 10.12688/f1000research.73820.3

**Published:** 2024-06-05

**Authors:** Nugrahanti Prasetyorini, Nabila Erina Erwan, Teguh Wahju Sardjono, Tatit Nurseta, Rudi Priyo Utomo, Rivo Yudhinata Brian Nugraha, Wike Astrid Cahayani, Ettie Rukmigarsari, Latania Naufa Arinugraha, Loeki Enggar Fitri

**Affiliations:** 1 Doctoral Program in Medical Science, Faculty of Medicine, Universitas Brawijaya, Malang, Indonesia; 2 Malaria Research Group, Faculty of Medicine, Universitas Brawijaya, Malang, Indonesia; 3 Department of Obstetrics & Gynecology, Faculty of Medicine Universitas Brawijaya/dr Saiful Anwar Hospital, Malang, Indonesia; 4 Master Program in Biomedical Science, Faculty of Medicine, Universitas Brawijaya, Malang, Indonesia; 5 Department of Parasitology, Faculty of Medicine, Universitas Brawijaya, Malang, Indonesia; 6 Department of Obstetrics & Gynecology, dr T.C. Hillers Regional Hospital, Maumere, Sikka Regency, NTT, Indonesia; 7 Department of Anatomy and Histology, Faculty of Medicine, Universitas Brawijaya, Malang, Indonesia; 8 Mathematics Education Study Program, Faculty of Teacher Training and Education, University of Islam Malang, Malang, Indonesia

**Keywords:** Placental malaria, Plasmodium vivax, LBW, HIF1-α, angiogenesis factors

## Abstract

**Background:**

Malaria in pregnancy leads to placental malaria. The primary pathogenesis of the complex fetal implications in placental malaria is tissue hypoxia due to sequestrations of *Plasmodium falciparum*-infected erythrocytes in the placenta. However, the pathomechanism of placental *Plasmodium vivax* infection has not been thoroughly investigated. Hypoxia-inducible factor-1α (HIF-1α) is a key transcriptional mediator of the response to hypoxic conditions, which interacts with the change and imbalances of many chemical mediators, including angiogenic factors, leading to fetal growth abnormality.

**Methods:**

This study was conducted cross-sectionally in Maumere, Sikka Regency, East Nusa Tenggara Province, previously known as one of the malaria endemic areas with a high incidence of low birth weight (LBW) cases. This study collected peripheral and umbilical blood samples and placental tissues from mothers who delivered their babies with LBW at the TC Hiller Regional Hospital. All of the blood samples were examined for parasites by microscopic and PCR techniques, while the plasma levels of VEGF, PlGF, VEGFR-1, VEGFR-2, and HIF-1α were determined using ELISA. The sequestration of infected erythrocytes and hemozoin was determined from placental histological slides, and the expression of placenta angiogenic factors was observed using the immunofluorescent technique.

**Results:**

In this study, 33 cases had complete data to be analyzed. Of them, 19 samples were diagnosed as vivax malaria and none of falciparum malaria. There were significant differences in Δ 10th percentile growth curve of baby's body weights and also all angiogenic factors in placental tissues {VEGF, PlGF, and VEGFR-1, VEGFR-2, and HIF-1α} between those infected and not infected cases (p<0.05), but not for VEGF and VEGFR-2 in the plasma.

**Conclusion:**

This study indicated that *Plasmodium vivax* sequestration may promote LBW through alterations and imbalances in angiogenic factors led by HIF-1α.

## Introduction

Malaria is still a major public health problem and the main cause of disease and death in developing countries ^1^. Young children and pregnant women, especially in the 1 ^st^ and 2 ^nd^ trimesters, are the populations most vulnerable to malaria infection ^1^. In malaria-endemic areas, at least 25% of pregnant women are infected with malaria, which accounts for 20% of maternal deaths ^2, 3^. Rijken *et al*. reported that around 75 million pregnant women in the Asia-Pacific region were exposed to *Plasmodium vivax* in 2007 ^2^. In 2019, for 33 countries in the World Health Organisation (WHO) African Region with moderate to high malaria transmission, it was estimated that 12 million (35%) of 33 million pregnancies were exposed to *Plasmodium falciparum* malaria ^4^. Globally, malaria causes more than 10,000 maternal deaths and 200,000 neonatal deaths annually, most of which are due to low birth weight (LBW) ^4– 6^. Pregnant women living in stable transmission areas are at risk for placental malaria, and pregnant women living in unstable areas have three times the risk of developing severe malaria than non-pregnant adult women living in the same areas ^2, 3^.

Indonesia is one of the countries in Southeast Asia with a relatively high number of malaria cases. In 2016, there were 218,480 positive cases of malaria, which decreased by almost half of the positive malaria cases in 2012 ^7^. The malaria parasites found were *Plasmodium falciparum* (62%) and *Plasmodium vivax* (37%). Based on WHO reported malaria cases by health facilities data in Indonesia (2016), there were 218,450 confirmed cases of malaria with a death rate of 161 cases ^8^. Papua, West Papua, and East Nusa Tenggara (NTT) are the three provinces with high malaria-endemic rates. Three regencies in NTT, which are highly endemic areas for malaria, are Sikka Regency, Lembata Regency, and Ngada Regency. Sikka Regency is located on Flores Island, borders East Flores Regency and Ende Regency. It covers 7,436.10 km ^2^ and had a population of 315,477, according to a 2010 survey ^9^. In 2008, 87,622 clinical malaria cases were reported in Sikka Regency ^10^. A previous study conducted on 92 babies born with low birth weight in T.C. Hillers Regional Hospital, Maumere, Sikka Regency, between December 2012 and December 2013 reported that 39 (42.4%) infants had congenital malaria, caused by *Plasmodium falciparum, Plasmodium vivax,* or mixed infection, of which 19 (48.7%) infants were asymptomatic, while the rest had sepsis, jaundice, and prematurity ^11^. This finding might emphasize that *Plasmodium vivax* also causes comparable effects, as *Plasmodium falciparum* has been linked to low birth weight and gestational malaria cases. Furthermore, the prevalence of instances among pregnant mothers may differ depending on the particular research location or endemic region in question.

Placental malaria is characterized by the accumulation of parasite-infected erythrocytes, mononuclear cells, and malaria pigment (hemozoin) in the placental blood vessels ^12^. Most reports of placental malaria are caused by infection with *Plasmodium falciparum*, which sequesters in the syncytiotrophoblast. This sequestration occurs by expressing *Plasmodium falciparum* erythrocyte membrane protein 1 (PfEMP1) surface protein, which specifically mediates the cytoadhesion of *Plasmodium falciparum*-infected erythrocytes to placental chondroitin sulfate A (CSA) located in the syncytiotrophoblast ^6, 13, 14^. This cytoadhesion and sequestration prevent the escape of circulating adult parasites, thereby avoiding the mechanism of clearance by complement and spleen. During the occurrence of placenta malaria, the Th1/Th2 balance shifts to the Th1 pathway, and there is an increase in the production of the pro-inflammatory cytokines, interferon-ϒ (IFN-ϒ) and tumor necrosis factor-α (TNF-α). This inflammatory response causes changes in the structure and function of the placenta, which are associated with poor pregnancy outcomes such as maternal anemia, prematurity, stunted fetal growth, and LBW ^15^. Placental malaria is a major cause of stunted fetal growth with poor pregnancy outcomes in the form of babies born with LBW ^16^.

Placenta malaria will evoke complement activation, both systemically and at the maternal-fetal interface in the placenta ^17^. There is an increase in the complement of anaphylatoxin C5a in circulating blood and placental tissue in mothers with placental malaria ^5^. Previous *in vitro* studies showed that *Plasmodium falciparum* glycosylphosphatidylinositol (PfGPI), together with C5a, increased pro-inflammatory milieu at the maternal-fetal interface in the form of increased cytokines and chemokines derived from monocytes ^18^. One of the consequences is the occurrence of dysregulation of angiogenic factors ^19^, which causes stunted fetal growth ^5, 6, 20, 21^. The local oxygen environment during pregnancy is one of the essential regulatory factors of angiogenesis. The main pathway of oxygen regulation of gene expression is hypoxia-inducible factor-1α (HIF-1α). Under hypoxic conditions, HIF-1α accumulation upregulates VEGF, which is a major proangiogenic factor directly. VEGF activity will induce the expression of vascular endothelial growth factor receptor-1 (VEGFR-1/Flt-1) and vascular endothelial growth factor receptor-2 (VEGFR-2/KDR). During hypoxia, VEGF will bind to receptors and stimulate capillary growth. Hypoxia-inducible factor-1α (HIF-1α) also increases the soluble anti-angiogenic factor Flt-1 (sFlt-1) ^22, 23^. High placental HIF-1α expression after the 1 ^st^ trimester of pregnancy is an abnormal condition that describes the occurrence of placental hypoxia due to inadequate placentation. Increased placental HIF-1α expression will cause an increase in placental sFlt-1 levels, which will be released into the maternal circulation. Soluble Flt-1 (sFlt-1) strongly binds free VEGF and PlGF, decreasing placental angiogenic activity. This condition is found in pregnancies with pre-eclampsia and IUGR ^24– 26^ and in placental malaria ^27^. Increased placental HIF-1α expression is also found in maternal anemia ^28^.

Carvalho *et al*., for the first time, demonstrated that *Plasmodium vivax*-infected erythrocytes (Pv-iEs) can perform *ex vivo* cytoadhesion on human lung endothelial cells (HLECs), Saimiri brain endothelial cells (SBECs), and placental cryosections both under static and flow conditions. This cytoadhesion is smaller than that of *Plasmodium falciparum*-infected erythrocytes (Pf-iEs) and is partly mediated by the VIR protein encoded by the *Plasmodium vivax* variant (vir) gene ^29^. Fernandez-Becerra *et al*. showed in their study that spleen-dependent *Plasmodium vivax* genes encode immunogenic proteins during infection, so it can be said that the spleen is an organ that plays a major role in expressing parasitic proteins involved in cytoadhesion ^30^. Pv-iEs binding to endothelial cells is mediated by glycosaminoglycans, namely chondroitin sulfate A (CSA) and hyaluronic acid (HA) ^29, 31^. Although all stages of the *Plasmodium vivax* were found in peripheral blood, only a few mature forms of schizonts were found. It is impossible to establish whether the disproportionate *Plasmodium vivax* parasitemia results from its sequestration in specific organs ^32^.

Data on the number of pregnancies infected with *Plasmodium vivax* have not been widely published. The mechanism and clinical implications of *Plasmodium vivax* infection in pregnancy are still unknown, possibly because it is considered to cause a milder clinical effect than *Plasmodium falciparum* infection ^33^. However, many studies have reported that vivax malaria in pregnancy is more associated with low birth weight and maternal anemia ^34, 35^ and may not be affected by parity because women living in areas with low malaria endemicity have low immunity to malaria ^36^. In this study, an *in vivo* study was conducted on pregnant women infected with malaria, emphasizing placental malaria. It aimed to determine the effect of placental and maternal plasma angiogenesis factors on the pathomechanism of LBW through HIF-1α regulation. This study used a peripheral blood sample to determine gestational malaria and a cord blood sample to determine placental malaria. Placental samples were also examined to identify the placenta's pathological properties and diagnose placental malaria. It is the first study of placental malaria angiogenesis factors in Indonesia using human placenta samples.

## Methods

### Ethics approval and consent

This research was conducted according to the guidelines of the Declaration of Helsinki and approved by the Faculty of Medicine Universitas Brawijaya Ethics Committee number 307/EC/KEPK/11/2018. Written informed consent, consisting of consent stating the baby's data to be used, was obtained from all subjects before they participated in this study.

### Characteristics of research participants

This study used a cross-sectional research design in T.C. Hillers Regional Hospital, Maumere, Sikka Regency, East Nusa Tenggara Province, a malaria-endemic area in Indonesia. This study used a purposive sampling technique in managing research participants. Thus, all of the subjects which met the inclusion criteria were included. This study was conducted for 10 months and the subjects were pregnant women who came to T.C. Hillers Regional Hospital in 2018 and met the inclusion criteria. The inclusion criteria were pregnant women who gave birth to babies with low birth weight (LBW) (< 2500 g) and small for gestational age (SGA) (birth weight < 10th percentile for gestational age at birth). Women who were primigravidae (being pregnant for the first time) and multigravidae (being pregnant more than once) were included in this study. Subjects were divided into two groups. They were (i) the case group, pregnant women who met the inclusion criteria and the malaria diagnosis criteria, and (ii) the control group, pregnant women who met the inclusion criteria and did not meet the malaria diagnosis criteria. All the participants were in term pregnancy, and the gestational age ranged from 37 to 42 weeks ^37^. Malaria cases were determined based on the discovery of *Plasmodium* parasites on examination of thin or thick blood smears and/or the discovery of *Plasmodium* DNA in maternal blood and or umbilical cord blood by polymerase chain reaction (PCR) examination, and or the discovery of *Plasmodium* parasites or hemozoin in the placenta.

### Diagnosis of placental malaria

The diagnosis of malaria case in this study was confirmed by the discovery of either the asexual form of *Plasmodium* on examination of thick or thin blood smears derived from maternal blood and or umbilical cord blood and *Plasmodium* DNA obtained by polymerase chain reaction (PCR) examination and finding of placental malaria, which is determined by placental tissue histopathological examination based on infected erythrocyte sequestration, monocyte infiltration, and hemozoin deposition ^38^. Hemozoin is a brown pigment crystal that forms in the digestive vacuole of *Plasmodium* as a product of hemoglobin catabolism ^39^. Hemozoin was seen as greenish-black or yellowish-brown granules using a light microscope and deposited intra or extra erythrocytes. Observations were made using a light microscope with 1000x magnification in 100 fields of view. Identification of infected erythrocytes was carried out by observing 100 fields of view. Hemozoin deposition was identified by observing 100 fields, and then the total number of hemozoin was calculated by dividing all fields by 100. The count includes hemozoin found within and outside infected erythrocytes, hemozoin attached to connective tissue, in the intervillous space (free hemozoin), or hemozoin inside free macrophages or macrophages covered by fibrin in the intervillous space.

### Definition of low birth weight

Low birth weight is a birth weight of less than 2500 g ^37^. In this study, what is meant by low birth weight is the difference between birth weight and the 10th percentile of the mean birth weight curve for boys and girls by gestational age from the reference curves of birth weight, length, and head circumference for gestational ages in Yogyakarta, Indonesia ^40^.

### Maternal peripheral blood sample preparation

In total, 10 milliliters of maternal venous blood were drawn from the median vein and put into a vacutainer containing anticoagulant (BD Vacutainer EDTA Tubes, 366643) for making blood smear preparations, plasma, and dried blood spots on filter paper. Microscopic examination was carried out on blood smear preparations. Dried blood spots were then used for PCR examination to confirm malaria diagnosis. Plasma was stored at 20°C and sent to Malang City (in collaboration with the Prodia Laboratory) in less than 48 hours. Using the ELISA method, these samples were then used to examine plasma levels of VEGF, PlGF, VEGFR-1 (sFlt-1), VEGFR-2, and HIF-1α.

### Umbilical artery blood sample preparation

In total, five milliliters of blood were drawn from the neonates' umbilical arteries and collected in an anticoagulant tube (BD Vacutainer EDTA Tubes, 367863) for hemoglobin and leukocyte count testing. Blood smears were made to identify malaria parasites, as well as dried blood spots on filter paper for PCR analysis.

### Parasitemia examination

The blood sample was dropped on an object glass, where a thin smear was made and dried. Furthermore, the smears were fixed evenly using absolute methanol and dried. The smears were stained with Giemsa solution (a mixture of Giemsa stain (Merck, HX612241) and Giemsa buffer (Bioanalytica, Indonesia) with a ratio of 1:9), then rinsed and dried. The thin smear slides were examined microscopically at 100 times magnification under the objective lens with immersion oil on at least 100 visual fields for each examination. To calculate the degree of parasitemia, 1000x magnification was carried out using a light microscope (Olympus Biological Microscope, CX23), counting the number of erythrocytes infected with malaria parasites per 1000 erythrocytes ^11^.

### Placental sampling for histopathological preparation

Samples obtained from the central part of the gross placental anatomy with a size of 2 cm and fixation with 10% neutral buffer formalin were then embedded into paraffin for immunofluorescence examination to determine the expression of VEGF, PlGF, VEGFR-1, VEGFR-2, and HIF-1α, the sequestration of infected erythrocytes and or hemozoin, as well as placental parasitemia. Placental histology preparations were made by cutting tissue that had previously been fixed with 10% neutral buffer formalin and inserted into a tissue cassette. Then, the samples were put into a basket and processed using a tissue processor tool (Thermo Scientific Microm STP 120 Spin Tissue Processor). The process of casting into paraffin blocks was done using a tissue embedding tool (Sakura Tissue-Tek TEC 5 Embedding Station). The paraffin blocks were cooled in a freezer before cutting with a microtome (Leica, RM2245). Then, the tissue was put into an incubator (Memmert, UN30) for 30 minutes in a 70–80°C temperature setting to maximize further deparaffination. Furthermore, after the tissue was removed from the incubator, deparaffination and Hematoxylin-Eosin staining were performed using the Tissue Tex DRS 2000 Multiple Slide Stainer tool.

### Immunofluorescence preparation

Immunofluorescence examination procedure on placental tissue was used to measure the placental expression of variables VEGF (anti-VEGFA antibody, SC 7269 FITC), PlGF (anti-PLGF antibody, SC 518003), VEGFR-1 (anti-VEGFR-1 antibody, SC 271789 PE), VEGFR-2 (anti-VEGFR-2 antibody, SC 6251 FITC), and HIF-1α (anti-HIF-1α antibody (SC 13515 PE). The immunofluorescence procedure was performed by single staining for HIF-1α and double staining for other variables. The immunofluorescence images were observed using a fluorescence microscope Olympus IX71 with 400x magnification. The results of the immunofluorescence images were then quantified using ImageJ 1.52p Fiji software. The parameter used was integrated density (IntDent), defined with the sum of the pixel value in selection (RawIntDen) multiplied by area in scaled units, then divided by area in pixel (RawInden * (Area in scaled units) / (Area in pixels)).

### Nested Polymerase Chain Reaction (PCR) examination to detect *Plasmodium falciparum* and *Plasmodium vivax* DNA

According to the manufacturer's instructions, DNA samples were obtained from blood sample extraction using PureLinkTM Genomic DNA Kits (Invitrogen, Carlsbad, California, USA). After purification, all samples were stored at -20°C till ready for the nested PCR. For the amplification of *Plasmodium* genus sequences, outer primer pairs (rPLU1 and rPLU5) were used. In the second reaction, two inner primers were used to detect *P. falciparum* (rFal1-rFal2) and *P. vivax* (rVIV1-rVIV2). PCR was performed using a Go Tag® Green Master Mix (Promega, Madison, Wisconsin, USA). 1 l template was treated with the following conditions: 1 l of each primer (10 M), 12.5 l of PCR master mix, and 9.5 l of double-distilled H _2_O _2_. The second nest reactions were carried out similarly using different primers. Sterile distilled water was used as a control ^11^.

### Enzyme-Linked Immunosorbent Assay (ELISA)

ELISA examination was performed to measure plasma levels of VEGF (human VEGF ELISA kit, Biolegend, Catalog No. 446507), PlGF (human PGF ELISA kit, bt-Lab, Catalog No. EO138Hu), VEGFR-1(sFlit1) (human VEGFR-1 ELISA kit, Invitrogen, REF#BMS268-3), VEGFR-2 (human VEGFR-1 ELISA kit, Invitrogen, REF#BMS2019), and HIF-1α (human HIF-1α ELISA kit, Invitrogen, REF#EHIF1A). The technique used was a quantitative sandwich enzyme immunoassay with the following procedure. The ELISA's standard was pipetted into a microplate that had been pre-coated with a specific antibody to the antigen according to the manufactured ELISA Kit. Each sample was pipetted into each well so the antigen would be bound to the immobilized antibody. Unbound materials were discarded. Then, a biotin-conjugated antibody specific to the antigen was added to the wells and washed. Afterwards, avidin-conjugated Horseradish Peroxidase (HRP) was added and washed to remove unbound materials. Staining was done by adding a solution substrate, incubating for 30 minutes, and adding a stop solution to stop the reaction. The preparations were read with a microplate reader (ZENIX-320).

### Neonatal examination

The obstetrician examined newborns for birth weight, length, head circumference, mid-upper arm circumference, chest circumference, Apgar score, placental size, and placental weight. The birth weight was measured immediately after birth using calibrated weighing scales. The birth length, head circumference, mid-upper arm circumference, and chest circumference were measured using a measuring tape. Apgar score was noted at the first minute of birth. The placental size was measured using a ruler, and the weight was measured using calibrated weighing scales.

### Statistical analysis

All statistical analyses were conducted using the SPSS 23 Statistic Program for Windows. The techniques for analyzing data include the normality test using the Shapiro-Wilk test to determine the normal data distribution. Comparative analysis between the case and control groups was carried out using a one-way ANOVA test for normally distributed data and the Kruskal Wallis test for non-normally distributed data to assess dysregulation of angiogenesis in the pathomechanism of low birth weight due to malaria-inhibited fetal growth in the placenta. The correlation test used was a Pearson test for normally distributed data and Spearman's rho test for non-normally distributed data to assess the correlation between the variables observed in the study. Path analysis was used to determine the conceptual research framework, i.e., whether the authors' concepts are relevant to the research findings. Data analysis was carried out with a confidence level of 95% and a degree of significance by p≤0.05.

## Results

### Subject characteristics

A total of 33 subjects met the inclusion criteria of this study Table 1. This study showed no statistically significant differences between the case and control groups in maternal age, gestational age, and maternal leukocyte count. In the case group, the age distribution of pregnant women is slightly lower than the age distribution of pregnant women in the control group, but this is not statistically different. Statistically significant differences were only found in maternal hemoglobin levels in the case group (p=0,00<α), lower than in the control group. The subject characteristics and distributions are shown in Table 1.

**Table 1.** Subject characteristics and distributions.

Characteristics	Controls (N=14) mean ± SD (min-max)	Cases (N=19) mean ± SD (min-max)	*p-value*
**Age (year)**	32.21±6.25 (20–38)	27.68±7.21 (19–39)	0.08
**Gestational Age (Weeks)**	38.64±0.84 (38–40)	39.47±1.39 (38–42)	0.08
**Haemoglobin Level (g dL ^-1^)**	11.10±1.20 (10.01–13.16)	9.88±0.62 (9.03–11.28)	**0.00**
**Leukocyte Number (10 ^3^ µL ^-1^)**	7796.79±2385.25 (4560–13890)	7622.11±2317.69 (4900–14200)	0.68

**Notes:** All data were analyzed with the Mann-Whitney test. The case group was pregnant mothers who were diagnosed with malaria (+); The control group was normal pregnant mothers/malaria (-) *SD = standard deviation.*

The laboratory tests were carried out to identify the control and case groups, as shown in Table 2.

**Table 2.** Laboratory examination results.

No	Examination	Controls (N=14)	Cases (N=19)	Notes
**1**	Thin/Thick Peripheral Blood Smear	26	7	*P. vivax*
**2**	Blood PCR from Mother	21	12	*P. vivax*
**3**	Cord Blood PCR	31	2	*P. vivax*
**4**	Placental Histopathological Examination	14	19	IE/hemozoin (+)

**Notes**: IE: *infected erythrocytes; PCR=polymerase chain reaction.*

**Figure 1.**
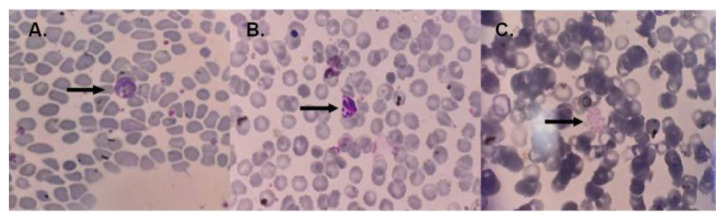
Blood smears from the malaria case group. In panel **A**, the arrow indicates the amoeboid form of *Plasmodium vivax*, panel **B** shows young schizonts of *Plasmodium vivax*, and panel **C** displays the mature schizont of *Plasmodium vivax.* The image size is the only modification made; no changes to brightness or contrast have been made. The examination was performed by using a light microscope (Olympus Biological Microscope, CX23) with 1000x magnification. The figures were captured using a mobile device, which could explain the absence of a scale bar.

**Figure 2.**
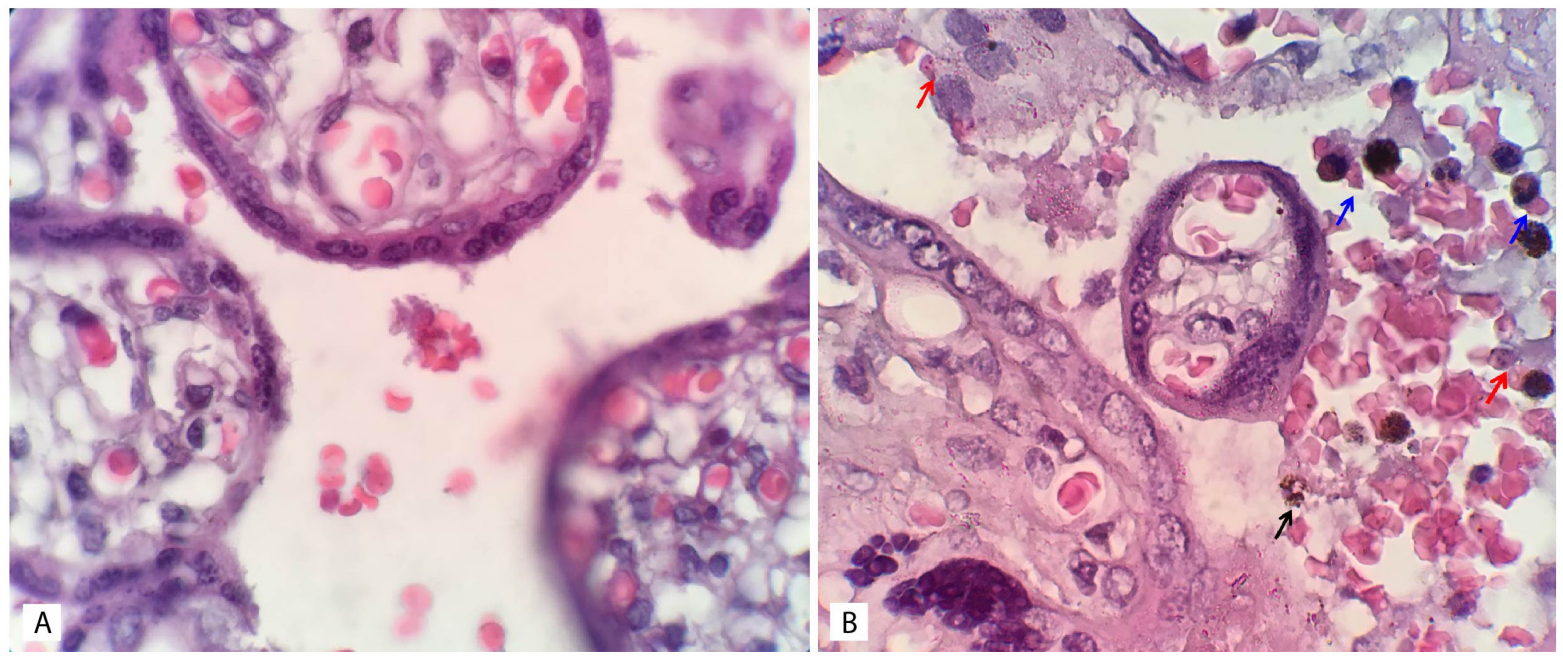
Histopathological examination of placental tissue. **A**: Normal placental tissue. **B**: Placental malaria. The red arrow indicates infected erythrocyte sequestration, the blue arrow indicates monocyte infiltration, and the black arrow indicates free hemozoin in the intervillous space. The image size is the only modification made; no changes to brightness or contrast have been made. The examination was performed by using a light microscope (Olympus Biological Microscope, CX23) with 1000x magnification. The figures were captured using a mobile device, which could explain the absence of a scale bar.

*Plasmodium vivax* was identified in all subjects of the case group, and no *Plasmodium falciparum* nor mixed infection was found. Histopathological examination of placental tissue from the case group showed sequestration of infected erythrocytes and/or hemozoin, followed by monocyte infiltration in the intervillous space in all samples. Two of the 19 malaria cases were identified from cord blood PCR. The examination of maternal peripheral blood smear can at least identify parasites compared to PCR and histopathological examination of the placenta, which is about 36.84%. The blood smears from the malaria case group are shown in Figure 1. Histopathological examination of placental tissue, which showed placental malaria compared to normal placental tissue, are shown in Figure 2.

The characteristics of the babies in this study were recorded, as shown in Table 3. There was a significant difference (p=0,00<α) in the mean Δ 10th percentile growth curve of birth weight between the case group (413.16±460,33 g) and the control group (-42.86±129.88 g). However, there was no significant difference (p=0.33>α) in the mean birth weight (g) between the case group (2189.47 ±182.06 g) and the control group (2239.29 ±190.85 g). The mean value of birth weight in the case group appears to be significantly smaller than the mean in the control group. It can be said that pregnant women with malaria are likely to give birth to a baby with a smaller birth weight than a baby born to a non-malaria pregnant woman.

**Table 3.** The results of the comparison test of baby characteristics.

Variables	Controls Mean ± deviation	Cases Mean ± deviation	*p-value*
**Δ 10 ^th^ percentile growth curve (g)**	-42.86±129.88	413.16±460.33	0.00 **
**Baby birthweight (g)**	2239.29 ±190.85	2189.47 ±182.06	0.33 **
**Body length (cm)**	41.86±2.91	42.95±3.58	0.45 **
**Head circumference (cm)**	27.21±1.05	27.47±1.26	0.50 **
**Upper arm circumference (cm)**	6.21±0.70	6.05±0.71	0.50 **
**Chest circumference (cm)**	20.71±1.33	21.00±1.89	0.53 **
**APGAR score**	9.14±0.36	9.16±0.60	0.85 **
**Placental size (cm ^3^)**	404.86±44.84	392.16±40.08	0.42 *
**Placental weight (g)**	457.14±43.22	450.00±57.73	0.52 **
**Hemoglobin level (g dL ^-1^)**	16.35±2.30	14.96±2.49	0.11 *
**Leukocyte number (10 ^3^ µL ^-1^)**	10.24±2.11	12.70±2.81	0.01 *

Note: *) comparative results on the free sample t-test **) comparative results on the Mann-Whitney test

### Association of angiogenic factors expression with birth weight

VEGF in the placenta of the case group was lower (64,404±28,942) than the control group (226,693±39,025). In both the case and control groups, VEGF expression was more abundant in the trophoblast cells of the placental villi than in the intervillous space. PlGF expression in the placenta of the case group was lower (22,814,440±9,497,663) than the control group (84,693,238±29,981,727). PlGF expression in the case group was abundant in trophoblast cells of the placental villi, whereas it was primarily found in the intervillous space in the control group. PlGF expression in the placenta of the case group was significantly lower than that of the control group.

**Figure 3.**
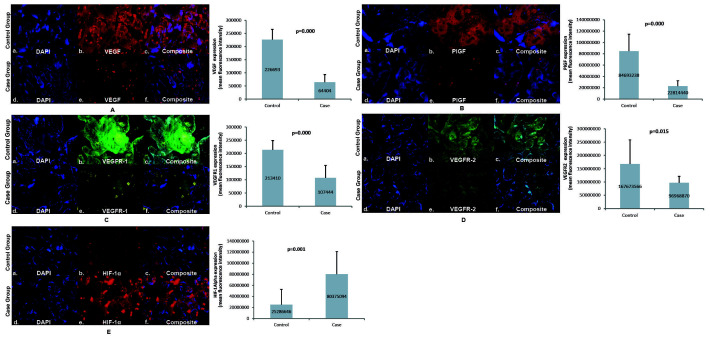
Angiogenic factors expression in placenta. Panel **A** to **E** shows the angiogenic expression in the placenta using immunofluorescence staining of VEGF, PlGF, VEGFR-1, VEGFR-2, and HIF-1α, respectively, with its semiquantitative analysis; VEGF: vascular endothelial growth factor; PlGF: placental growth factor; VEGFR-1: vascular endothelial growth factor receptor-1; VEGFR-2: vascular endothelial growth factor receptor-2; HIF-1α: hypoxia-inducible factor-1α. The examination was conducted using a fluorescence microscope Olympus IX71 with 400x magnification. The immunofluorescence figures were captured using a mobile device, which could explain the absence of a scale bar.

The expression of VEGFR-1 in the placenta of the case group was lower (107,444±46,696) than the control group (213,410±35,251). VEGFR-1 expression was more abundant in trophoblast cells in the placental villi than in the intervillous space in the case and control groups. The expression of VEGFR-1 in the case group's placenta was significantly lower than that of the control group. VEGFR-2 expression in the placenta of the case group was lower (96,968,870±24,300,623) than the control group (167,673,566±90,824,566). VEGFR-2 expression was abundant in trophoblast cells in the placental villi in the case and control groups. The expression of VEGFR-2 in the case group's placenta was significantly lower than that of the control group. The expression of HIF-1α in the placenta of the case group was higher (80,375,094±40,647,360) than the control group (25,286,646±27,238,953). In the case and control groups, HIF-1α expression was more commonly found in the trophoblast cells of the placental villi. HIF-1α expression in the placenta of the case group was significantly higher than that of the control group, as seen in Figure 3.

The path analysis test results showed a significant direct effect (p=0.04) on HIF-1α expression on VEGF expression with an effect coefficient of -0.55. Likewise, there was a significant direct effect (p=0.03) of HIF-1α expression on PlGF expression with an effect coefficient of -0.60. VEGF expression had no direct effect on VEGFR-1 expression (p=0.89) but had a direct effect on VEGFR-2 expression (p=0.00) with a coefficient of effect of 0.75. There was a significant direct effect (p=0.00) on PlGF expression on VEGFR-1 expression, with an effect coefficient of 0.94. VEGFR-1 expression had no direct effect on birth weight (p=0.46), but VEGFR-2 expression had a significant direct effect (p=0.02) on birth weight with an effect coefficient of 0.74.

### Comparison test of angiogenic factor in the placenta and maternal plasma

**Figure 4.**
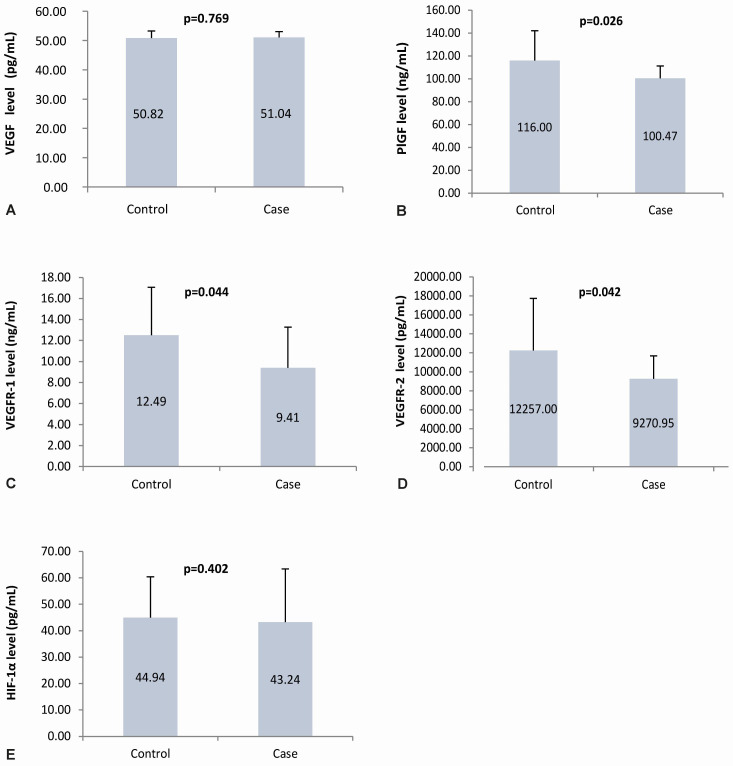
Angiogenic factors level in maternal plasma. Panel **A** to **E** shows the angiogenic factors level in the maternal plasma using ELISA of VEGF, PlGF, VEGFR-1, VEGFR-2, and HIF-1α, respectively; VEGF: vascular endothelial growth factor; PlGF: placental growth factor; VEGFR-1: vascular endothelial growth factor receptor-1; VEGFR-2: vascular endothelial growth factor receptor-2; HIF-1α: hypoxia-inducible factor-1α.

The mean maternal plasma VEGF levels in the case and control groups were almost the same; statistical results showed no statistically significant difference (p=0.77). The mean maternal plasma PlGF, VEGFR-1, and VEGFR-2 levels in the case group were lower than in the control group. Statistically, there is a significant difference (p=0.03, 0.04, 0.04, respectively). Similar to the VEGF plasma level, the HIF-1α plasma levels in the case and control groups had values that were not much different, which showed no significant difference (p=0.40). The angiogenic factor levels in the case and control group are presented in Figure 4.

### Comparison test of the placenta and maternal plasma data

There was a significant difference in the mean expression of VEGF, PlGF, VEGFR-1, and VEGFR-2 placenta between the case and control groups. The mean value of placental VEGF, PlGF, VEGFR-1, and VEGFR-2 expression in the case group was lower than the mean in the control group. In addition, there was a significant difference in the mean placental HIF-1α expression between the case and the control groups. However, in contrast to other angiogenic factors, the mean placental HIF-1α expression in the case group was higher than the mean placental HIF-1α expression in the control group. The comparison of angiogenic factor expression in the placenta is shown in Table 4.

**Table 4.** The comparison test of angiogenic factors in the placenta and maternal plasma between the case and control group.

Variable	Controls (n=14) Mean ± SD		Cases (n=19) Mean ± SD		*p-value*	
	Placenta (Mean fluorescence intensity)	Plasma	Placenta (Mean fluorescence intensity)	Plasma	Placenta	Plasma
**VEGF**	226,693 ± 39,025	50.82 ± 2.41 (pg/mL)	64,404 ± 28,942	51.04 ± 1.99 (pg/mL)	0.00 *	0.77 *
**PlGF**	84,693,238 ± 29,981,727	116.00 ± 26.19 (ng/mL)	22,814,440 ± 9,497,663	100.47 ± 10.72 (ng/mL)	0.00 *	0.03 *
**VEGFR-1**	213,410 ± 35,251	12.49 ± 4.57 (ng/mL)	107,444 ± 46,695	9.41 ± 3.86 (ng/mL)	0.00 *	0.04 *
**VEGFR-2**	167,673,566 ± 90,824,566	12257 ± 5482 (pg/mL)	96,968,870 ± 24,300,623	9271 ± 2419 (pg/mL)	0.02 **	0.04 *
**HIF-1α**	25,286,646.1±27,238,953	44.94 ± 15.49 (pg/mL)	80,375,094 ± 40,647,360	43.24 ± 20.19 (pg/mL)	0.00 **	0.40 **

Notes: *) T-test **) Mann-Whitney test *SD=standard deviation.*

There was no significant difference in the mean VEGF plasma levels between the case and control groups. The mean value of VEGF plasma levels in the case group was slightly higher than the average VEGF level in the control group. However, this increase was not statistically significant. Similar to VEGF, the mean plasma levels of HIF-1α between case and control groups did not show a significant difference. However, HIF-1α in the case group was slightly lower than the mean HIF-1α expression in the control group. The plasma levels of PlGF, VEGFR-1, and VEGFR-2 between the case and control groups showed significant differences. The mean levels of PlGF, VEGFR-1, and VEGFR-2 in the case group were lower than in the control group. The comparison of maternal plasma angiogenic factor levels is shown in Table 4.

## Discussion

All malaria-positive samples in this study were identified using maternal peripheral blood smear examination or PCR examination caused by *Plasmodium vivax* ( Table 2). Microscopic examination of blood smears only detected 7 of the 19 positive samples (36.84%). On the other hand, 12 blood samples were detected positively by PCR examination (63.16%). The results showed that PCR is more sensitive than blood smear examination. Malaria detection using microscopic examination could only detect *Plasmodium* at 20 parasites/μL levels. It was influenced by the experience of the observer and the quality of staining blood smears. PCR is the most sensitive method because it can detect 2–6 parasites/μL ^41– 44^. It was surprising that all the histopathological preparations of 19 samples showed pigment/hemozoin depositions with or without infected erythrocytes or monocytes accumulation, indicating that all of the samples had already been infected as active acute, active chronic, and past infection ^38, 45– 47^. In this study, all positive samples did not show signs and symptoms of malaria, so all positive samples were subclinical infections.

Investigation of acute infection through maternal peripheral blood smear examination cannot fully describe the history of maternal exposure to malaria during pregnancy. The absence of parasitemia on peripheral blood smear examination does not always represent a malaria-free placental infection ^46^. In this study, acute, chronic, and past malaria infection in pregnancy may be related to the adverse consequences of placental histopathological changes. In cases of subclinical malaria, placental histopathological changes might be found due to most of the sub-populations of parasites sequestered in several organs that serve as reservoirs of the parasite, such as the spleen and placenta, causing other inflammatory reactions ^42, 46, 48, 49^.

### Characteristics of subjects and their distribution

*Characteristics of mothers.* This study showed significant differences in the mean hemoglobin levels in women infected with *Plasmodium vivax* during pregnancy compared to those not infected. At the same time, gestational age, maternal age, and leukocyte count showed no significant difference. The average hemoglobin level of pregnant women infected with malaria was 1.22 g/dL lower than the control group (mean ± standard deviation [SD]; 9.88±0.62 vs 11.10±1.20, p=0.00). This finding aligned with several previous studies, which also showed that vivax malaria infection is associated with maternal anemia, abortion, premature birth, congenital malaria, and other severe complications ^36, 50– 54^. Single *Plasmodium vivax* infection during pregnancy had a significant association with a twofold increased risk of maternal anemia compared to the group of mothers who did not experience malaria infection ^36, 55^. The occurrence of maternal anemia is closely correlated with nutritional status ^36^. However, one of the other risk factors that can increase anemia in pregnant women with *Plasmodium vivax* infection is young age pregnancy ^56^. In this study, the mean age of pregnant women in the case group was lower than the control group (mean ± SD; 27.68 ± 7.21 vs. 32.21 ± 6.25, p = 0.08); there was no significant difference. This factor may be considered one of the critical risk factors in the subsequent study involving many subjects.

The parity distribution in the case group was mainly primiparity (11/19; 57.9%), while in the control group, the highest number was mothers with a third parity (6/14; 42.9%). This result has a distribution pattern similar to Bardaji *et al.*. In their study, most malaria pregnant women were primigravida, and malaria prevalence tended to decrease as gravidity increased ^35^. In a study of Karen women in Thailand involving a comparable number of participants, *Plasmodium vivax* was more common in primigravida than in multigravida, and this was associated with anemia and an increased risk of LBW ^51^.

Both case and control groups showed no significant difference (mean ± SD: 39.47±1.39 vs. 38.64±0.84, p=0.078) in gestational age, but all participants gave birth at term. In the study of Bardaji *et al*., it was found that the average gestational age was 38.6 weeks, with 12.6% of all participants giving birth prematurely (<37 weeks) ^35^. From the results of their study, it cannot be concluded that there is a related relationship because it requires a joint analysis with other factors such as the severity of malaria infection, the onset of malaria infection, and pregnancy outcomes, which in this case includes the baby's birth weight. As in this study, all infants were born at term but had a significant effect of small for gestational age with malaria infection status in the form of the chronic active or previous history of malaria.

***Characteristics of babies.*** This study showed a significant difference in the mean Δ 10th percentile growth curve of birth weight between the case groups. However, there were no significant differences in the average birth weight of the baby, the length of the baby's birth weight, head circumference, upper arm circumference, chest circumference, Apgar score, placental weight, placenta size, and hemoglobin levels. However, the baby's mean hemoglobin level in the case group was 1.39 g/dL lower than the control group. The result was consistent with a previous study, which reported that a single episode of *P. vivax* infection during pregnancy was associated with a significant reduction in neonatal birth weight and maternal hemoglobin levels ^33^. The results of the meta-analysis study also stated that there was an increased risk of 1-2 times the occurrence of LBW in pregnant women infected with malaria compared to those who were not infected ^57^.

Meanwhile, babies in both the case and control groups were included in LBW criteria, so it is unknown whether the leading cause of LBW in the case group is influenced by the main factor of *Plasmodium vivax* infection, as in the previous studies ^33, 56^. According to Bardaji *et al*., there is no direct relationship between *Plasmodium vivax* infection and an increased risk of LBW, but they stated that *Plasmodium vivax* infection during pregnancy is closely associated with maternal anemia, where this condition may harm the health of the newborn ^35^. In addition, maternal anemia is a risk factor for the occurrence of LBW with an odds ratio (OR) value of 1.23 (95% confidence interval [CI]: 1.06–1.43) ^58^.

The results showed significant differences in the LBW leukocyte count parameters in the case and control groups (mean ± SD: 12.70 × 10 ^3^±2.81/μL vs. 10.24 × 10 ^3^±2.11/μL, p<0.05). In general, the leukocyte count values in newborns can range from 10,000–26,000/μL, which is also highly dependent on the time of sampling ^59^. Meanwhile, the results of the leukocyte count in newborns at Bantul Hospital, Indonesia, in 195 infants showed an average leukocyte count of 14.62 × 10 ^3^±3.49/μL ^60^. The leukocyte count in newborns in the case and control groups showed values within normal limits, with the leukocyte count in the case group significantly higher than the control group. Several factors can increase the leukocyte count in newborns, including infection, inflammation, medication history, and stress ^61– 63^.

### Sequestration of hemozoin, infected erythrocytes, and monocytes in histopathology result

Sequestration is the attachment of infected erythrocytes to host cells associated with severe malaria, such as cerebral malaria and malaria in pregnancy. Sequestration occurs in the capillaries and post-capillary venules of specific organs such as the brain, lungs, and placenta. Sequestration correlates with mechanical obstruction of blood flow in the microvasculature and the activation of vascular endothelial cells, leading to pathological outcomes. Some parasitic and host proteins as ligands and receptors for sequestration have been identified and explored further ^64^.

The sequestration phenomenon occurred in *Plasmodium falciparum* infection ^45, 65, 66^. Interestingly, none of the subjects in this study were infected with *Plasmodium falciparum*. However, hemozoin depositions were found in all 19 positive samples, while *Plasmodium vivax*-infected erythrocyte sequestrations were found in some samples. The result indicates that the *Plasmodium vivax* could cause sequestration of infected erythrocytes. Submicroscopic placental infection of *Plasmodium vivax* confirmed by placental histopathology means that *Plasmodium vivax* can be selectively sequestered in the placenta even at low densities in peripheral blood ^67^. The finding of *Plasmodium vivax*-infected erythrocytes sequestration was also mentioned by Carvalho *et al*. through an *ex vivo* study, which found that *Plasmodium vivax*-infected erythrocytes could adhere to the placental tissue. This study also demonstrated the cytoadherence of *Plasmodium vivax*-infected erythrocytes on endothelial cells by *in vitro* approach. It was known to have the same strength as cytoadherence of *Plasmodium falciparum*-infected erythrocytes ^29^. Toda *et al*. found that *Plasmodium vivax*-infected reticulocytes could bind to human spleen fibroblasts (hSF). The binding involves the transcription factor of the NF-kB signaling pathway by plasma-derived extracellular vesicles that act as cargo for the *Plasmodium vivax* parasite. This process facilitates the expression of ICAM-1 on the surface of hSF as a receptor for *Plasmodium vivax* sequestration ^49^. Other evidence supporting placental malaria due to other non-falciparum *Plasmodium* species is a submicroscopic infection of *Plasmodium malariae* and *Plasmodium ovale* in the placenta confirmed by PCR of placental blood samples. Plasmodium malariae infection was detected higher in placental blood samples than in peripheral blood samples, indicating a possibility of *Plasmodium malariae* binding affinity to the placenta. However, the study of non- *falciparum* placental infection was not supported by histopathological examination of the placenta ^68^.

Malaria infection causes changes in the placenta structure in the form of hemozoin deposition and an increase in monocyte infiltration in the intervillous space, which interferes with maternal-fetal circulation ^46^. Sequestration of infected erythrocytes, hemozoin deposition, and monocyte infiltration in placental histopathology are the main signs of placental malaria ^12, 69, 70^. The pathomechanism of sequestration of infected erythrocytes in the placenta is clearly described in *Plasmodium falciparum* infection. However, *Plasmodium vivax*, which has been considered to cause a mild latent infection than *Plasmodium falciparum*, has also been reported to cause placental malaria. Histopathological changes of placental malaria due to *Plasmodium falciparum* can show a different representation of placental malaria due to *Plasmodium vivax*. However, these differences have not yet been delineated ^46^. The histopathological appearance of the placenta due to *Plasmodium vivax* infection is similar to that of *Plasmodium falciparum*. *Plasmodium vivax* has also demonstrated the sequestration mechanism of infected erythrocytes in placental tissue structures ^47, 71^. These findings are consistent with the results of this study.

Several mechanisms can explain the *Plasmodium vivax*-infected erythrocytes sequestration phenomenon. First, the *Plasmodium vivax*-infected erythrocyte sequestration can occur under low shear stress conditions in the placental intervillous space and causes a local inflammatory process of the placenta ^31^. Second, *Plasmodium vivax* lacks surface proteins, such as *Plasmodium falciparum* Erythrocyte Membrane Protein-1 (PfEMP-1) called Variant Surface Antigen-2-CSA (VAR2CSA) for adhesion and sequestration of CSA in the placental intervillous space ^72, 73^. However, the *Plasmodium vivax* genome contains a subtelomeric multigene family called VIR proteins. VIR proteins can mediate the adhesion of infected erythrocytes to ICAM-1 by VIR14 and the CSA by VIR2 and VIR24. The bindings are speculative and require further research ^71, 72, 74^. The discovery of VIR binding in ICAM-1 and CSA proved the presence of *Plasmodium vivax* sequestration in the previous study ^29^. The third mechanism that can explain the sequestration of *Plasmodium vivax* is the formation of rosettes by interacting with the Glycophorin C receptor present on normal erythrocytes, which has the adhesive power as in *Plasmodium falciparum*. However, further research is needed to determine the specific mechanism of rosette formation ^71, 75, 76^.

On histopathological examination of the placenta, it was found that pigment/hemozoin depositions were trapped in the fibrin or freely circulating in the intervillous space and monocyte infiltration in all 19 positive samples. This finding indicates that the mother had experienced malaria infection during her pregnancy ^45, 77^. The presence of pigment/hemozoin and monocyte depositions in the placenta suggests malaria infection does not occur in late pregnancy. The infection process has occurred long enough to cause deposition and accumulation of inflammatory cells in the placenta acquired early in pregnancy or before pregnancy ^67, 68^. However, the time required for placental malaria's appearance is unknown and requires further research.

### The placental expression and plasma level of VEGF, PlGF, VEGFR-1, VEGFR-2, and HIF-1α in placenta malaria

***Vascular Endothelial Growth Factor (VEGF).*** In this study, VEGF expression in the placenta of the case group was lower than in the control group. In the case and control groups, VEGF expression was more abundant in trophoblast cells in the placental villi than in the intervillous space. VEGF expression in the case group placenta was significantly lower than in the control group (p=0.00). Expression of VEGF placental IUGR was inconsistent between several studies that have been carried out. The results of this study are consistent with several studies, which reported a decrease in VEGF expression accompanied by increased placental PlGF expression of IUGR ^78, 79^. Several studies reported an increase in the expression of VEGF, VEGFA, bFGF, and eNOS in the IUGR placenta due to placental hypoxia ^80– 82^. However, another study concluded no decrease in VEGF expression of IUGR compared to normal placentas ^83, 84^. In addition, VEGF expression and plasma VEGF levels could not predict late-onset pre-eclampsia, small gestational age (SGA), or premature delivery ^85^.

Plasma VEGF levels in the case group were lower and not significantly different from the control group in this study (p=0.77). The findings were consistent with several studies, which found that maternal plasma VEGF levels of IUGR and SGA did not show significant differences compared to pre-eclampsia ^86^ or normal pregnancy ^87^. Moreover, Tang *et al*. reported that maternal plasma VEGF levels in pregnancies with pre-eclampsia were lower than in normal pregnancies and were associated with impaired fetal growth ^88^. However, Boras *et al*. found that maternal free plasma levels of VEGF and sFlt-1 were higher in IUGR than in normal pregnancies ^89^.

Besides regulating development and vascular remodeling during placentation, VEGF, PlGF, and Angiopoietin are also involved in trophoblast invasion. Failure of trophoblast invasion and remodeling of the spiral arteries causes placental hypoxia, which involves pregnancy complications such as pre-eclampsia and IUGR ^90^, causing the fetus to experience hypoxia. At low oxygen concentrations, there should be an increase in VEGF expression. The mechanism that can explain the decrease in placental VEGF expression in this study is increased soluble VEGFR-1 (sFlt-1) in the placental intervillous space. sFlt-1 is a form of the VEGFR-1 gene physiologically secreted by the placenta. sFlt-1 physiologically binds to free VEGF and PlGF with solid affinity and inhibits their binding to VEGFR-1 and VEGFR-2 receptors, decreasing placental VEGF and PlGF expression. HIF-1α regulates placental sFlt-1 expression; under conditions of placental hypoxia, there will be an increase in placental sFlt-1 expression ^91, 92^. The sFlt-1 expression was also reported to increase in IUGR placentas ^93, 94^. An *in vivo* study in transgenic mice by Vogtmann *et al*. found that an increase in placental sFlt1 would disrupt the vascular endothelial growth factor signaling pathway, resulting in decreased expression of VEGFA, VEGFB, and PlGF and also increased the expression of band and mRNA of Caspase-9 ^95^. Placental hypoxia will increase placental VEGF expression ^90, 91^; however, the source of circulating VEGF is not only from the placenta but also from other organs ^96^, which can explain why the plasma levels of VEGF in the case group and the control group were not different.

This study's finding of decreased placental VEGF expression can describe early-onset IUGR because placental hypoxia correlated with placental HIF-1α expression. However, histo-morphological studies of placental villi were not performed to confirm the dominance of branching angiogenesis in placental villi. Early-onset IUGR in this study can be caused by malaria infection before pregnancy or early pregnancy ^45^, as evidenced in this study.

***Placental Growth Factor (PlGF).*** The placenta is the only organ that produces PlGF ^96^. PlGF significantly functions in the activation, proliferation, and migration of endothelial cells and trophoblast invasion into the maternal spiral arteries ^97^. In this study, the expression of PlGF in the placenta in the case group was lower than in the control group (p=0.00). In the case group, PlGF expression was more abundant in trophoblast cells in the placental villi, whereas in the control group, it was primarily found in the intervillous space. Studies on PlGF expression in placental IUGR have also shown inconsistent results. The results of this study support previous studies that reported that placental PlGF expression in severe IUGR was significantly lower than in controls, and inhibition of PlGF/Flt-1 signalling will interfere with trophoblast proliferation and migration ^97– 99^. However, Alahakoon *et al*. found an increase in PlGF and KDR expression in IUGR and pre-eclampsia placentas ^84^. It explained that hyperoxia conditions in IUGR due to impaired oxygen extraction to the fetus increase PlGF expression and decrease placental VEGF expression, with the consequence of reduced capillary branching and terminal villi resulting in placental dysfunction ^90, 91, 100, 101^.

Placental hypoxia conditions that occur early in pregnancy decrease placental PlGF production by the syncytiotrophoblast, so PlGF expression and PlGF levels in maternal circulation decrease ^90, 91, 98^. In this study, the plasma levels of PlGF in the case group were significantly lower than in the control group (p=0.03). Previous studies have also shown that plasma or plasma PlGF levels are lower in pregnancies with IUGR ^93, 102, 103^, pregnancies with pre-eclampsia ^95, 104– 106^, and pregnancies with pre-eclampsia and IUGR ^86, 88, 94, 107– 113^. Decreased maternal plasma PlGF levels in pregnancy complications due to defective placentation are associated with increased production of sFlt-1 in the ischemic placenta, which is then secreted into the maternal circulation ^102, 104^. Furthermore, significantly lower PlGF levels in the case group indicate that the causes of IUGR in the two groups may be different. Malaria infection in the placenta causes the activation of complement C5a, which will stimulate the accumulation of monocytes to release sFlt-1 ^18^, explaining that the plasma levels of PlGF in the case group were significantly lower than the control group.

***Vascular Endothelial Growth Factor Receptor (VEGFR)-1 and VEGFR-2.*** This study showed that VEGFR-1 expression in the placenta was lower in the case group than in the control group (p=0.00). VEGFR-1 expression was more abundant in trophoblast cells in the placental villi than in the intervillous space, both in the case and control group. VEGFR-1 is a receptor for VEGF and PlGF. Helske *et al*. found that VEGFR-1 expression was increased in preeclamptic and IUGR placentas compared with normal placentas. The upregulated expression is not exactly found on all preeclamptic samples but may be associated with hypoxia and abnormal function of the placenta ^114^. However, the down-regulation of both receptors was also reported during mid-pregnancy with IUGR ^115^.

In line with VEGFR-1, the expression of VEGFR-2 in the case group's placenta was lower than in the control group (p=0.02). In the case and control groups, VEGFR-2 expression was more abundant in trophoblast cells in the placental villi. VEGFR-2 is a significant receptor for VEGF and plays a role in endothelial proliferation and normal vascular formation. In the placenta, the role of VEGFR-2 is to change trophoblasts into intravascular trophoblasts and form spiral arteries. VEGFR-2 is expressed in low amounts under hypoxic conditions because oxygen levels regulate VEGFR-2 expression. Placental VEGFR-2 and sFlt-1 expression was also reported to decrease in cases of pre-eclampsia ^93, 116^.

In this study, plasma levels of VEGFR-1 in the case group were lower and significantly different from the control group (p=0.04). Anna *et al*. found that serum VEGFR-1 concentrations in women with IUGR were decreased, along with a decrease in PlGF. At low oxygen concentrations, PlGF and VEGFR-1 levels decrease ^117^. Besides that, serum levels of VEGFR-2 in the case group were lower and significantly different from the control group (p=0.04). Chaiworapongsa *et al*. found soluble VEGFR-2 (sVEGFR2) concentration increased in women with preeclamptic pregnancies and interpreted that sVEGFR-2 in maternal plasma could reflect endothelial cell function ^118^.

***Hypoxia-Inducible Factor-1α (HIF-1α).*** This study revealed that the expression of HIF-1α in the case group's placenta was higher than in the control group (p=0.00). In the case group and the control group, HIF-1α expression was more commonly found in trophoblast cells in the placental villi. There are few studies in humans examining HIF-1α expression in the placenta of pregnancies with complications such as pre-eclampsia, IUGR, and SGA. The results of this study support an *in vivo* study on the IUGR mice model conducted by Robb *et al*., who found an increase in HIF-1α expression in the placenta ^119^.

In this study, plasma levels of HIF-1α in the case group were lower and not significantly different from the control group (p=0.40). So far, studies of HIF-1α have been mainly carried out in placental tissue, as in the discussion on placental HIF-1α expression above. Ashur-Fabian *et al*. found elevated HIF-1α and p21 mRNA expression in all pregnancy plasma samples with hypoxia and IUGR and recommended them as markers for hypoxic pregnancy and/or IUGR ^120^. In pregnancy, HIF-1α is expressed in the placenta early in normal pregnancy and throughout pregnancy under hypoxic conditions ^121^. Those explain no statistically significant difference in plasma HIF-1α levels in the case and control groups.

Under normoxic conditions, HIF-1α will undergo rapid degradation so that it is considered inactive. HIF-1α will regulate genes that control cell growth, differentiation, and metabolism at low oxygen levels. The active form of HIF-1α continuously exposed to cultured cells inhibited trophoblast differentiation. In placental IUGR, impaired invasion and remodeling of the spiral arteries result in placental ischemia throughout pregnancy because of the increased expression of HIF-1α. The increase in placental HIF-1α expression in this study represents early-onset IUGR. However, this study has not determined whether placental hypoperfusion causes hypoxia as the main factor driving the increase in HIF-1α because other factors, such as reactive oxygen species (ROS) and inflammation induced by nuclear factor kappa (NF-κB), can also modulate HIF-1α accumulation.

### Association between low birth weight and VEGF, PlGF, VEGFR-1, VEGFR-2, HIF-1α expression in placenta

Path analysis revealed the effect of placental angiogenesis and HIF-1α transcription factors on LBW. This analysis showed that the signaling pathway from HIF-1α to placental VEGF expression, then from placental VEGF expression to placental VEGFR-2 expression, and from placental VEGFR-2 expression to LBW showed statistically significant results. In contrast, HIF-1α could not cause a direct effect on LBW (p=0.88). Placental VEGF expression significantly affected placental VEGFR-1 expression. The signaling pathway of HIF-1α significantly affected placental PlGF expression, and then placental PlGF expression significantly affected placental VEGFR-1 expression, but this pathway did not significantly affect the incidence of LBW (p=0.50).

VEGF and VEGFR regulate vasculogenesis during early embryogenesis and angiogenesis in later stages of pregnancy. VEGF binds to VEGFR-1/Flt-1 and VEGFR-2/KDR, which is essential in physiological and pathological angiogenesis ^122^. PlGF binds to the VEGFR-1 receptor and plays a role in angiogenesis in the later stages of pregnancy ^123^. VEGFR-2 is the primary receptor mediating the proangiogenic effects of VEGF ^124^. sFlt1, the free form of Flt-1, can bind strongly to VEGFA, PlGF, and VEGFB. Because the trophoblast lies between the maternal and fetal vascular systems, it is thought that sFlt1 functions as a separator between the maternal and fetal circulations in the placenta by suppressing excessive angiogenesis and abnormal vascular permeability. Therefore, sFlt1 levels are maintained in the suitable range under physiological conditions. The increased expression of sFLT-1, triggered by hypoxic conditions, will bind to VEGF, causing disturbances in vasculogenesis and angiogenesis, leading to obstetric complications. Several studies have reported overexpression of sFlt1 and decreased expression of VEGFA in preeclamptic patients ^125^.

In placental malaria infection, Plasmodium-infected erythrocytes accumulate in the intervillous space of the placenta. Several histopathological characteristics of placental malaria, including thickening of the basement membrane and infiltration of monocytes in the intervillous space of the placenta (intervillositis), can increase the resistance to oxygen transport across the placenta. Accumulation of inflammatory cells and infected erythrocytes can cause placental hypoxia due to oxygen consumption by these cells and decreased blood perfusion due to reduced effective surface area for feto-maternal exchange. Such placental dysfunction can result in stunted fetal growth, characterized by low birth weight babies ^27^.

Increased expression of HIF-1α is a marker of placental hypoxia, which should increase the expression of VEGF, VEGFR-1, and VEGFR-2. Decreased VEGFR, VEGFR-1, and VEGFR-2 expression is possible through increased sFlt-1 binding to VEGF with strong affinity. sFlt-1 of the placenta increases its expression under hypoxic conditions ^91, 92^. Further studies are needed to prove this.

The pathway analysis results indicate a dysregulation of the angiogenic factor VEGF and its receptor VEGFR-2 due to the regulation of HIF-1α in placental malaria on the incidence of LBW. It can be interpreted that the occurrence of LBW in placental malaria is due to the influence of angiogenesis factors VEGF and VEGFR-2, which are regulated by HIF-1α and are more significant than LBW in non-malaria cases.

### Association between low birth weight and hemoglobin, PlGF levels, VEGFR-1 levels, and VEGFR-2 levels in plasma

The effect of plasma angiogenesis and HIF-1α transcription factors on the incidence of LBW was observed using binary logistic regression as bivariate analysis. Based on the observational test of data in maternal plasma, four parameters were significantly different between the case group and the control group: hemoglobin, PlGF levels, VEGFR-1 levels, and VEGFR-2 levels. It showed that hemoglobin levels were the most significant (p=0.01) in influencing the incidence of LBW, followed by plasma levels of PlGF (p=0.02) and plasma levels of VEGFR-1 (p=0.05). The plasma levels of VEGFR-2 did not affect the incidence of LBW (p=0.13).

The hematological effect of *Plasmodium vivax* malaria is anemia with its consequent increased morbidity and mortality and more frequent blood transfusions. Although the parasitemia of *Plasmodium vivax* malaria is lower than that of *Plasmodium falciparum* malaria, it can cause severe anemia, as in *Plasmodium falciparum* malaria. Anemia in *Plasmodium vivax* malaria can be explained by two mechanisms. First, production/transfer of infected and uninfected erythrocytes are more in *Plasmodium vivax* malaria, with 34 uninfected erythrocytes for one infected erythrocyte (in *Plasmodium falciparum* malaria, the ratio is 8:1). Second, *Plasmodium vivax*-infected erythrocytes undergo more rapid deformity limiting erythrocytes that are expelled through the microvessels of the spleen during the 'spleen clearance' phase. In addition to these two mechanisms, activation of the immune system due to *Plasmodium vivax* infection increases the detection and removal of abnormal infected and uninfected erythrocytes ^55^.

In this study, maternal anemia significantly affected the incidence of LBW. Several publications also reported the same thing. *Plasmodium vivax* malaria infection in pregnant women in Bolivia was more at risk of anemia and giving birth to low birth weight babies than pregnant women who were not infected ^36^ as well as in a multicenter study in an area of low malaria transmission ^35^ and Mangaluru, India ^126^.

Based on the above discussion, in this study, it can be concluded that angiogenesis factors and HIF-1α transcription factors in the placenta or local environment play a more critical role in the incidence of low birth weight than those in the systemic. Those indicated that there is dysregulation of placental angiogenesis factors VEGF, PlGF, and their receptors VEGFR-1 and VEGFR-2, which is triggered by placental hypoxia conditions (marked by increased placental HIF-1α expression) during placental *Plasmodium vivax* infection. Further research with an increased sample size is needed to reveal this conclusion.

## Data Availability

Figshare: HIF-1α REGULATED PATHOMECHANISM OF LOW BIRTH WEIGHT THROUGH ANGIOGENESIS FACTORS IN PLACENTAL *Plasmodium vivax* INFECTION. https://doi.org/10.6084/m9.figshare.16577468 ^127^. This project contains the following underlying data: Raw Data.xlsx (ELISA Graph and Raw Data, Immunofluorescence Graph and Raw Data, Subject Characteristics, and Baby's Characteristics Data) Figure 1A (Blood Smear Figure 1A) Figure 1B (Blood Smear Figure 1B) Figure 1C (Blood Smear Figure 1C) Figure 2A (VEGF immunofluorescence) Figure 2B (PlGF immunofluorescence) Figure 2C (VEGFR-1 immunofluorescence) Figure 2D (VEGFR-2 immunofluorescence) Figure 2E (HIF-α immunofluorescence) Figure 3 (Angiogenic Factor Expression Graph) Figure 4 (Angiogenic Factor Level in Maternal Plasma Graph) PCR Images 1–4 (Raw Gel Images of PCR test from maternal (1–3) and cord (4) blood samples) Data are available under the terms of the Creative Commons Zero "No rights reserved" data waiver (CC0 1.0 Public domain dedication).

## References

[1] Centers for Disease Control and Prevention (CDC): Malaria's Impact Worldwide. CDC,2021. Reference Source

[2] Rijken MJ McGready R Boel ME : Malaria in pregnancy in the Asia-Pacific region. *Lancet Infect Dis.* 2012;12(1):75–88. 10.1016/S1473-3099(11)70315-2 22192132

[3] Kovacs SD Rijken MJ Stergachis A : Treating severe malaria in pregnancy: a review of the evidence. *Drug Saf.* 2015;38(2):165–81. 10.1007/s40264-014-0261-9 25556421 PMC4328128

[4] WHO: World Malaria Report 2020. WHO,2020;73:1–4. Reference Source

[5] Conroy AL Silver KL Zhong K : Complement activation and the resulting placental vascular insufficiency drives fetal growth restriction associated with placental malaria. *Cell Host Microbe.* 2013;13(2):215–26. 10.1016/j.chom.2013.01.010 23414761

[6] Sharma L Shukla G : Placental malaria: a new insight into the pathophysiology. *Front Med (Lausanne).* 2017;4:117. 10.3389/fmed.2017.00117 28791290 PMC5524764

[7] Ministry of Health of the Republic of Indonesia: Malaria Elimination in Indonesia.2017.

[8] WHO Report: World Malaria Report 2017. World Health Organization,2017. Reference Source

[9] Sikka Regency Government: Profile of Sikka Regency.2017. Reference Source

[10] Kazwaini M Laumalay HM Prasetyawan FS : SISTEM SURVEILANS MALARIA DI PROVINSI NUSA TENGGARA TIMUR.2011. Reference Source

[11] Fitri LE Jahja NE Huwae IR : Congenital malaria in newborns selected for low birth-weight, anemia, and other possible symptoms in maumere, Indonesia. *Korean J Parasitol.* 2014;52(6):639–44. 10.3347/kjp.2014.52.6.639 25548415 PMC4277026

[12] Muehlenbachs A Nabasumba C McGready R : Artemether-lumefantrine to treat malaria in pregnancy is associated with reduced placental haemozoin deposition compared to quinine in a randomized controlled trial. *Malar J.* 2012;11(1): 150. 10.1186/1475-2875-11-150 22554092 PMC3487992

[13] Pehrson C Mathiesen L Heno KK : Adhesion of *Plasmodium falciparum* infected erythrocytes in *ex vivo* perfused placental tissue: a novel model of placental malaria. *Malar J.* 2016;15(1): 292. 10.1186/s12936-016-1342-2 27230523 PMC4881162

[14] Ayres Pereira M Mandel Clausen T Pehrson C : Placental sequestration of *Plasmodium falciparum* malaria parasites is mediated by the interaction between VAR2CSA and chondroitin sulfate A on syndecan-1. *PLoS Pathog.* 2016;12(8): e1005831. 10.1371/journal.ppat.1005831 27556547 PMC4996535

[15] Talundzic E : Plasticity and diversity of the *Plasmodium falciparum* placental malaria antigen VAR2CSA. University of Georgia,2013. Reference Source

[16] Schlaudecker EP Munoz FM Bardají A : Small for gestational age: Case definition & guidelines for data collection, analysis, and presentation of maternal immunization safety data. *Vaccine.* 2017;35(48 Pt A):6518–6528. 10.1016/j.vaccine.2017.01.040 29150057 PMC5710996

[17] Ngai M Weckman AM Erice C : Malaria in pregnancy and adverse birth outcomes: new mechanisms and therapeutic opportunities. *Trends Parasitol.* 2020;36(2):127–37. 10.1016/j.pt.2019.12.005 31864896

[18] Conroy A Serghides L Finney C : C5a enhances dysregulated inflammatory and angiogenic responses to malaria *in vitro*: potential implications for placental malaria. *PLoS One.* 2009;4(3): e4953. 10.1371/journal.pone.0004953 19308263 PMC2655724

[19] McDonald CR Tran V Kain KC : Complement activation in placental malaria. *Front Microbiol.* 2015;6:1460. 10.3389/fmicb.2015.01460 26733992 PMC4685051

[20] Weckman AM Ngai M Wright J : The impact of infection in pregnancy on placental vascular development and adverse birth outcomes. *Front Microbiol.* 2019;10:1924. 10.3389/fmicb.2019.01924 31507551 PMC6713994

[21] Singh PP Bhandari S Sharma RK : Association of angiopoietin dysregulation in placental malaria with adverse birth outcomes. *Dis Markers.* 2020;2020: 6163487. 10.1155/2020/6163487 32399088 PMC7201683

[22] Nevo O Soleymanlou N Wu Y : Increased expression of sFlt-1 in *in vivo* and *in vitro* models of human placental hypoxia is mediated by HIF-1. *Am J Physiol Regul Integr Comp Physiol.* 2006;291(4):1085–93. 10.1152/ajpregu.00794.2005 16627691 PMC6428068

[23] Rath G Aggarwal R Jawanjal P : HIF-1 alpha and placental growth factor in pregnancies complicated with pre-eclampsia: a qualitative and quantitative analysis. *J Clin Lab Anal.* 2016;30(1):75–83. 10.1002/jcla.21819 25545166 PMC6807228

[24] Tal R Shaish A Barshack I : Effects of hypoxia-inducible factor-1alpha overexpression in pregnant mice: possible implications for pre-eclampsia and intrauterine growth restriction. *Am J Pathol.* 2010;177(6):2950–62. 10.2353/ajpath.2010.090800 20952590 PMC2993274

[25] Chen DB Zheng J : Regulation of placental angiogenesis. *Microcirculation.* 2014;21(1):15–25. 10.1111/micc.12093 23981199 PMC5589442

[26] Zimna A Kurpisz M : Hypoxia-Inducible factor-1 in physiological and pathophysiological angiogenesis: applications and therapies. *Biomed Res Int.* 2015;2015: 549412. 10.1155/2015/549412 26146622 PMC4471260

[27] Boeuf P Tan A Romagosa C : Placental hypoxia during placental malaria. *J Infect Dis.* 2008;197(5):757–65. 10.1086/526521 18279052 PMC2760295

[28] Michalitsi V Dafopoulos K Gourounti K : Hypoxia-inducible factor-1α (HIF-1α) expression in placentae of women with iron deficiency anemia and β-thalassemia trait. *J Matern Neonatal Med.* 2015;28(4):470–4. 10.3109/14767058.2014.921672 24803010

[29] Carvalho BO Lopes SCP Nogueira PA : On the cytoadhesion of *Plasmodium vivax*-infected erythrocytes. *J Infect Dis.* 2010;202(4):638–47. 10.1086/654815 20617923

[30] Fernandez-Becerra C Bernabeu M Castellanos A : *Plasmodium vivax* spleen-dependent genes encode antigens associated with cytoadhesion and clinical protection. *Proc Natl Acad Sci U S A.* 2020;117(23):13056–65. 10.1073/pnas.1920596117 32439708 PMC7293605

[31] Chotivanich K Udomsangpetch R Suwanarusk R : *Plasmodium vivax* adherence to placental glycosaminoglycans. *PLoS One.* 2012;7(4): e34509. 10.1371/journal.pone.0034509 22529919 PMC3328474

[32] Costa FTM Avril M Nogueira PA : Cytoadhesion of *Plasmodium falciparum*-infected erythrocytes and the infected placenta: a two-way pathway. *Braz J Med Biol Res.* 2006;39(12):1525–36. 10.1590/s0100-879x2006001200003 17160261

[33] Pincelli A Neves PAR Lourenço BH : The Hidden Burden of *Plasmodium vivax* malaria in pregnancy in the Amazon: an observational study in Northwestern Brazil. *Am J Trop Med Hyg.* 2018;99(1):73–83. 10.4269/ajtmh.18-0135 29741155 PMC6085809

[34] Rayis DA Ahmed MA Omer EM : *Plasmodium vivax* malaria among pregnant women in Eastern Sudan. *Asian Pacific J Trop Dis.* 2016;6(6):421–3. 10.1016/S2222-1808(15)61058-1

[35] Bardají A Martínez-Espinosa FE Arévalo-Herrera M : Burden and impact of *Plasmodium vivax* in pregnancy: a multi-centre prospective observational study. *PLoS Negl Trop Dis.* 2017;11(6):1–22. 10.1371/journal.pntd.0005606 28604825 PMC5481034

[36] Brutus L Santalla J Schneider D : *Plasmodium vivax* malaria during pregnancy, Bolivia. *Emerg Infect Dis.* 2013;19(10):1605–11. 10.3201/eid1910.130308 24050302 PMC3810741

[37] American College of Obstetricians and Gynecologists: Practice bulletin no. 134: fetal growth restriction. *Obs Gynecol.* 2013;121(5):1122–33. 10.1097/01.AOG.0000429658.85846.f9 23635765

[38] Rogerson SJ Hviid L Duffy PE : Malaria in pregnancy: pathogenesis and immunity. *Lancet Infect Dis.* 2007;7(2):105–17. 10.1016/S1473-3099(07)70022-1 17251081

[39] Olivier M Van Den Ham K Shio MT : Malarial pigment hemozoin and the innate inflammatory response. *Front Immunol.* 2014;5:25–10. 10.3389/fimmu.2014.00025 24550911 PMC3913902

[40] Haksari EL Lafeber HN Hakimi M : Reference curves of birth weight, length, and head circumference for gestational ages in Yogyakarta, Indonesia. *BMC Pediatr.* 2016;16(1): 188. 10.1186/s12887-016-0728-1 27871318 PMC5117525

[41] Moody A : Rapid diagnostic tests for malaria parasites. *Clin Microbiol Rev.* 2002;15(1):66–78. 10.1128/CMR.15.1.66-78.2002 11781267 PMC118060

[42] Harris I Sharrock WW Bain LM : A large proportion of asymptomatic *Plasmodium* infections with low and sub-microscopic parasite densities in the low transmission setting of Temotu Province, Solomon Islands: challenges for malaria diagnostics in an elimination setting. *Malar J.* 2010;9(1): 254. 10.1186/1475-2875-9-254 20822506 PMC2944341

[43] Golassa L Enweji N Erko B : Detection of a substantial number of sub-microscopic *Plasmodium falciparum* infections by polymerase chain reaction: a potential threat to malaria control and diagnosis in Ethiopia. *Malar J.* 2013;12(1): 352. 10.1186/1475-2875-12-352 24090230 PMC3850638

[44] Berzosa P De Lucio A Romay-Barja M : Comparison of three diagnostic methods (microscopy, RDT, and PCR) for the detection of malaria parasites in representative samples from equatorial Guinea. *Malar J.* 2018;17(1): 333. 10.1186/s12936-018-2481-4 30223852 PMC6142353

[45] Rogerson SJ Pollina E Getachew A : Placental monocyte infiltrates in response to *Plasmodium falciparum* malaria infection and their association with adverse pregnancy outcomes. *Am J Trop Med Hyg.* 2003;68(1):115–9. 10.4269/ajtmh.2003.68.1.0680115 12556159

[46] Parekh FK Davison BB Gamboa D : Placental histopathologic changes associated with subclinical malaria infection and its impact on the fetal environment. *Am J Trop Med Hyg.* 2010;83(5):973–80. 10.4269/ajtmh.2010.09-0445 21036823 PMC2963955

[47] López-Guzmán C Carmona-Fonseca J : Submicroscopic placental malaria: Histopathology and expression of physiological process mediators. *Rev Peru Med Exp Salud Publica.* 2020;37(2):220–8. 10.17843/rpmesp.2020.372.4759 32876209

[48] Chaikitgosiyakul S Rijken MJ Muehlenbachs A : A morphometric and histological study of placental malaria shows significant changes to villous architecture in both *Plasmodium falciparum* and *Plasmodium vivax* infection. *Malar J.* 2014;13(1):1–13. 10.1186/1475-2875-13-4 24386908 PMC3900675

[49] Toda H Diaz-Varela M Segui-Barber J : Plasma-derived extracellular vesicles from *Plasmodium vivax* patients signal spleen fibroblasts via NF-kB facilitating parasite cytoadherence. *Nat Commun.* 2020;11(1): 2761. 10.1038/s41467-020-16337-y 32487994 PMC7265481

[50] Rivera AJ Rivera LL Dubon JM : Effect of *Plasmodium vivax* malaria on perinatal health. *Rev Honduras Pediátrica.* 1993;16:7–10.

[51] Nosten F Rogerson SJ Beeson JG : Malaria in pregnancy and the endemicity spectrum: what can we learn? *Trends Parasitol.* 2004;20(9):425–32. 10.1016/j.pt.2004.06.007 15324733

[52] Kochar DK Saxena V Singh N : *Plasmodium vivax* malaria. *Emerg Infect Dis.* 2005;11(1):132–4. 10.3201/eid1101.040519 15705338 PMC3294370

[53] Rodriguez-Morales AJ Sanchez E Vargas M : Pregnancy outcomes associated with *Plasmodium vivax* malaria in Northeastern Venezuela. *Am J Trop Med Hyg.* 2006;74(5):755–7. 10.4269/ajtmh.2006.74.755 16687675

[54] Carvalho BO Matsuda JS Luz SL : Gestational malaria associated to *Plasmodium vivax* and *Plasmodium falciparum* placental mixed-infection followed by foetal loss: a case report from an unstable transmission area in Brazil. *Malar J.* 2011;10(1): 178. 10.1186/1475-2875-10-178 21708032 PMC3141593

[55] Douglas NM Anstey NM Buffet PA : The anaemia of *Plasmodium vivax* malaria. *Malar J.* 2012;11: 135. 10.1186/1475-2875-11-135 22540175 PMC3438072

[56] Poespoprodjo JR Fobia W Kenangalem E : Adverse pregnancy outcomes in an area where multidrug-resistant *Plasmodium vivax* and *Plasmodium falciparum* infections are endemic. *Clin Infect Dis.* 2008;46(9):1374–81. 10.1086/586743 18419439 PMC2875100

[57] Anggara FY Rahardjo SS Murti B : Meta-Analysis: The Effect of Malaria Infection on the Incidence of Low Birth Weight. *J Matern Child Heal.* 2020;5(5):549–62. Reference Source

[58] Figueiredo ACMG Gomes-Filho IS Silva RB : Maternal anemia and low birth weight: a systematic review and meta-analysis. *Nutrients.* 2018;10(5):601. 10.3390/nu10050601 29757207 PMC5986481

[59] Milcic TL : The complete blood count. *Neonatal Netw.* 2010;29(2):109–15. 10.1891/0730-0832.29.2.109 20211833

[60] Hariningrum A : Rentang Nilai Hitung Darah Lengkap Bayi Baru Lahir di RSU PKU Muhammadiyah Bantul. Universitas Gadjah Mada,2014. Reference Source

[61] Manroe BL Weinberg AG Rosenfeld CR : The neonatal blood count in health and disease. I. Reference values for neutrophilic cells. *J Pediatr.* 1979;95(1):89–98. 10.1016/s0022-3476(79)80096-7 480023

[62] Weinberg AG Rosenfeld CR Manroe BL : Neonatal blood cell count in health and disease. II. Values for lymphocytes, monocytes, and eosinophils. *J Pediatr.* 1985;106(3):462–6. 10.1016/s0022-3476(85)80681-8 4038739

[63] De Winter JP Van Bel F : The effect of glucocorticosteroids on the neonatal blood count. *Acta Paediatr Scand.* 1991;80(2):159–62. 10.1111/j.1651-2227.1991.tb11827.x 2035306

[64] Franke-Fayard B Fonager J Braks A : Sequestration and tissue accumulation of human malaria parasites: can we learn anything from rodent models of malaria? *PLoS Pathog.* 2010;6(9): e1001032. 10.1371/journal.ppat.1001032 20941396 PMC2947991

[65] Ismail MR Ordi J Menendez C : Placental pathology in malaria: a histological, immunohistochemical, and quantitative study. *Hum Pathol.* 2000;31(1):85–93. 10.1016/s0046-8177(00)80203-8 10665918

[66] Suguitan AL Jr Leke RG Fouda G : Changes in the levels of chemokines and cytokines in the placentas of women with *Plasmodium falciparum* malaria. *J Infect Dis.* 2003;188(7):1074–82. 10.1086/378500 14513430

[67] Mayor A Bardají A Felger I : Placental infection with *plasmodium vivax*: a histopathological and molecular study. *J Infect Dis.* 2012;206(12):1904–10. 10.1093/infdis/jis614 23053630 PMC6592414

[68] Doritchamou JYA Akuffo RA Moussiliou A : Submicroscopic placental infection by non- *falciparum Plasmodium* spp. *PLoS Negl Trop Dis.* 2018;12(2): e0006279. 10.1371/journal.pntd.0006279 29432484 PMC5825172

[69] Carmona-Fonseca J Arango E Maestre A : Placental malaria in Colombia: histopathologic findings in *Plasmodium vivax* and *P. falciparum* infections. *Am J Trop Med Hyg.* 2013;88(6):1093–101. 10.4269/ajtmh.12-0363 23546807 PMC3752808

[70] Souza RM Ataíde R Dombrowski JG : Placental histopathological changes associated with *Plasmodium vivax* infection during pregnancy. *PLoS Negl Trop Dis.* 2013;7(2): e2071. 10.1371/journal.pntd.0002071 23459254 PMC3573078

[71] Totino PR Lopes SC : Insights into the cytoadherence phenomenon of *Plasmodium vivax*: the putative role of phosphatidylserine. *Front Immunol.* 2017;8: 1148. 10.3389/fimmu.2017.01148 28979260 PMC5611623

[72] Requena P Rui E Padilla N : *Plasmodium vivax* VIR proteins are targets of naturally-acquired antibody and T cell immune responses to malaria in pregnant women. *PLoS Negl Trop Dis.* 2016;10(10): e0005009. 10.1371/journal.pntd.0005009 27711158 PMC5053494

[73] Zakama AK Ozarslan N Gaw SL : Placental malaria. *Curr Trop Med Rep.* 2020;7(4):162–171. 10.1007/s40475-020-00213-2 32953387 PMC7493061

[74] Bernabeu M Lopez FJ Ferrer M : Functional analysis of *Plasmodium vivax* VIR proteins reveals different subcellular localizations and cytoadherence to the ICAM-1 endothelial receptor. *Cell Microbiol.* 2012;14(3):386–400. 10.1111/j.1462-5822.2011.01726.x 22103402

[75] Marín-Menéndez A Bardají A Martínez-Espinosa FE : Rosetting in *plasmodium vivax*: a cytoadhesion phenotype associated with anaemia. *PLoS Negl Trop Dis.* 2013;7(4): e2155. 10.1371/journal.pntd.0002155 23593522 PMC3617122

[76] Albrecht L Lopes SCP da Silva ABIE : Rosettes integrity protects *Plasmodium vivax* of being phagocytized. *Sci Rep.* 2020;10(1): 16706. 10.1038/s41598-020-73713-w 33028898 PMC7541459

[77] Mens PF Bojtor EC Schallig HDFH : Molecular interactions in the placenta during malaria infection. *Eur J Obstet Gynecol Reprod Biol.* 2010;152(2):126–32. 10.1016/j.ejogrb.2010.05.013 20933151

[78] Khaliq A Li XF Shams M : Localization of placenta Growth Factor (PIGF) in human term placenta. *Growth Factors.* 1996;13(3–4):243–50. 10.3109/08977199609003225 8919031

[79] Lyall F Young A Boswell F : Placental expression of vascular endothelial growth factor in placentae from pregnancies complicated by pre-eclampsia and intrauterine growth restriction does not support placental hypoxia at delivery. *Placenta.* 1997;18(4):269–76. 10.1016/s0143-4004(97)80061-6 9179920

[80] Chen Q Schlichtherle M Wahlgren M : Molecular aspects of severe malaria. *Clin Microbiol Rev.* 2000;13(3):439–50. 10.1128/CMR.13.3.439 10885986 PMC88942

[81] Kinzler W Kizhner O Peltier M : 402: Role of angiogenesis-related genes in fetal growth restriction. *Am J Obstet Gynecol.* 2008;199(6):S122. 10.1016/j.ajog.2008.09.431

[82] Szentpéteri I Rab A Kornya L : Gene expression patterns of Vascular Endothelial Growth Factor (VEGF-A) in human placenta from pregnancies with intrauterine growth restriction. *J Matern Fetal Neonatal Med.* 2013;26(10):984–9. 10.3109/14767058.2013.766702 23350655

[83] Tse JY Lao TT Chan CC : Expression of Vascular Endothelial Growth Factor in third-trimester placentas is not increased in growth-restricted fetuses. *J Soc Gynecol Investig.* 2001;8(2):77–82. 10.1177/107155760100800203 11336877

[84] Alahakoon TI Zhang W Arbuckle S : Reduced angiogenic factor expression in intrauterine fetal growth restriction using semiquantitative immunohistochemistry and digital image analysis. *J Obstet Gynaecol Res.* 2018;44(5):861–72. 10.1111/jog.13592 29392826

[85] Andraweera PH Dekker GA Roberts CT : The Vascular Endothelial Growth Factor family in adverse pregnancy outcomes. *Hum Reprod Update.* 2012;18(4):436–57. 10.1093/humupd/dms011 22495259

[86] Afzal I : Master of philosophy the levels of angiogenic and anti-angiogenic molecule concentrations in pregnancy based disorders in the maternal and fetal.2012.

[87] Wallner W Sengenberger R Strick R : Angiogenic growth factors in maternal and fetal serum in pregnancies complicated by intrauterine growth restriction. *Clin Sci (Lond).* 2007;112(1):51–7. 10.1042/CS20060161 16928195

[88] Tang Y Ye W Liu X : VEGF and sFLT-1 in serum of PIH patients and effects on the foetus. *Exp Ther Med.* 2019;17(3):2123–8. 10.3892/etm.2019.7184 30867699 PMC6396009

[89] Borras D Perales-Puchalt A Ruiz Sacedón N : Angiogenic growth factors in maternal and fetal serum in pregnancies complicated with intrauterine growth restriction. *J Obstet Gynaecol.* 2014;34(3):218–20. 10.3109/01443615.2013.834304 24484391

[90] Gourvas V Dalpa E Vrachnis N : Placental angiogenesis and fetal growth restriction. From Preconception to Postpartum.2012;179. 10.5772/37879

[91] Arroyo JA Winn VD : Vasculogenesis and angiogenesis in the IUGR placenta. *Semin Perinatol.* Elsevier,2008;32(3):172–7. 10.1053/j.semperi.2008.02.006 18482617

[92] Tang L He G Liu X : Progress in the understanding of the etiology and predictability of fetal growth restriction. *Reproduction.* 2017;153(6):R227–40. 10.1530/REP-16-0287 28476912

[93] Nevo O Many A Xu J : Placental expression of soluble fms-like tyrosine kinase 1 is increased in singletons and twin pregnancies with Intrauterine Growth Restriction. *J Clin Endocrinol Metab.* 2008;93(1):285–92. 10.1210/jc.2007-1042 17956955

[94] Tsatsaris V Goffin F Munaut C : Overexpression of the soluble Vascular Endothelial Growth Factor receptor in preeclamptic patients: pathophysiological consequences. *J Clin Endocrinol Metab.* 2003;88(11):5555–63. 10.1210/jc.2003-030528 14602804

[95] Vogtmann R Kühnel E Dicke N : Human sFLT1 leads to severe changes in placental differentiation and vascularization in a transgenic hsFLT1/rtTA FGR mouse model. *Front Endocrinol (Lausanne).* 2019;10:165. 10.3389/fendo.2019.00165 30949132 PMC6437783

[96] Cerdeira AS Karumanchi SA : Angiogenic factors in preeclampsia and related disorders. *Cold Spring Harb Perspect Med.* 2012;2(11): a006585. 10.1101/cshperspect.a006585 23125198 PMC3543100

[97] Joó JG Rigó J Jr Börzsönyi B : Placental gene expression of the Placental Growth Factor (PlGF) in intrauterine growth restriction. *J Matern Fetal Neonatal Med.* 2017;30(12):1471–5. 10.1080/14767058.2016.1219993 27483982

[98] Wu WB Xu YY Cheng WW : Decreased PGF may contribute to trophoblast dysfunction in fetal growth restriction. *Reproduction.* 2017;154(3):319–29. 10.1530/REP-17-0253 28676532

[99] Ravikumar G Mukhopadhyay A Mani C : Placental expression of angiogenesis-related genes and their receptors in IUGR pregnancies: correlation with fetoplacental and maternal parameters. *J Matern Fetal Neonatal Med.* 2020;33(23):3954–61. 10.1080/14767058.2019.1593362 30922130

[100] Ahmed A Perkins J : Angiogenesis and intrauterine growth restriction. *Baillieres Best Pract Res Clin Obstet Gynaecol.* 2000;14(6):981–98. 10.1053/beog.2000.0139 11141345

[101] Burton GJ Charnock-Jones DS Jauniaux E : Regulation of vascular growth and function in the human placenta. *Reproduction.* 2009;138(6):895–902. 10.1530/REP-09-0092 19470597

[102] Benton SJ McCowan LM Heazell AEP : Placental Growth Factor as a marker of fetal growth restriction caused by placental dysfunction. *Placenta.* 2016;42:1–8. 10.1016/j.placenta.2016.03.010 27238707

[103] Komwilaisak R Tangkiratichai P : Maternal serum angiogenic growth factors in intrauterine growth restriction versus normal pregnancies. *J Med Assoc Thai.* 2017;100(2):119–24. 29916230

[104] Maynard SE Venkatesha S Thadhani R : Soluble Fms-like Tyrosine Kinase 1 and endothelial dysfunction in the pathogenesis of Pre-eclampsia. *Pediatr Res.* 2005;57(5 Pt 2):1R–7R. 10.1203/01.PDR.0000159567.85157.B7 15817508

[105] Vrachnis N Kalampokas E Sifakis S : Placental Growth Factor (PlGF): a key to optimizing fetal growth. *J Matern Fetal Neonatal Med.* 2013;26(10):995–1002. 10.3109/14767058.2013.766694 23330778

[106] Rădulescu C Bacârea A Huțanu A : Placental growth factor, soluble fms-like tyrosine kinase 1, soluble endoglin, IL-6, and IL-16 as biomarkers in Preeclampsia. *Mediators Inflamm.* 2016;2016: 3027363. 10.1155/2016/3027363 27799724 PMC5069373

[107] Bersinger NA Ødegård RA : Serum levels of macrophage colony stimulating, vascular endothelial, and Placenta Growth Factor in relation to later clinical onset of pre-eclampsia and a small-for-gestational age birth. *Am J Reprod Immunol.* 2005;54(2):77–83. 10.1111/j.1600-0897.2005.00290.x 16105099

[108] Karumanchi SA Epstein FH : Placental ischemia and soluble fms-like tyrosine kinase 1: cause or consequence of pre-eclampsia? *Kidney Int.* 2007;71(10):959–61. 10.1038/sj.ki.5002281 17495934

[109] Romero R Nien JK Espinoza J : A longitudinal study of angiogenic (Placental Growth Factor) and anti-angiogenic (soluble endoglin and soluble vascular endothelial growth factor receptor-1) factors in normal pregnancy and patients destined to develop pre-eclampsia and deliver a small for gestational age neonate. *J Matern Fetal Neonatal Med.* 2008;21(1):9–23. 10.1080/14767050701830480 18175241 PMC2587364

[110] Ghosh SK Raheja S Tuli A : Can maternal serum Placental Growth Factor estimation in early second trimester predict the occurrence of early onset pre-eclampsia and/or early onset intrauterine growth restriction? a prospective cohort study. *J Obstet Gynaecol Res.* 2013;39(5):881–90. 10.1111/jog.12006 23496304

[111] Laskowska M Laskowska K Oleszczuk J : aVEGF-A and its Soluble Receptor Type 1 (sVEGFR-1, sFlt-1) Concentrations in Pregnancies with Intrauterine Growth Restriction in the Presence or Absence of Preeclampsia. *Res J Pharm Biol Chem Sci.* 2015;6(2):319–25. Reference Source

[112] Chang YS Chen CN Jeng SF : The sFlt-1/PlGF ratio as a predictor for poor pregnancy and neonatal outcomes. *Pediatr Neonatol.* 2017;58(6):529–33. 10.1016/j.pedneo.2016.10.005 28571908

[113] Naimi AA Schmidt-fittschen M Herzeg A : Is there any association between the angiogenic factors sflt-1 / plgf and intrauterine growth restriction in patients with preeclamsia? *J Womens Health Gyn.* 2019;6(3):1–7. Reference Source

[114] Helske S Vuorela P Carpén O : Expression of Vascular Endothelial Growth Factor Receptors 1, 2 and 3 in placentas from normal and complicated pregnancies. *Mol Hum Reprod.* 2001;7(2):205–10. 10.1093/molehr/7.2.205 11160848

[115] Regnault TR de Vrijer B Galan HL : The relationship between transplacental O _2_ diffusion and placental expression of PlGF, VEGF and their receptors in a placental insufficiency model of Fetal Growth Restriction. *J Physiol.* 2003;550(Pt 2):641–56. 10.1113/jphysiol.2003.039511 12740423 PMC2343042

[116] Nevo O Lee DK Caniggia I : Attenuation of VEGFR-2 expression by sFlt-1 and low oxygen in human placenta. *PLoS One.* 2013;8(11): e81176. 10.1371/journal.pone.0081176 24260556 PMC3834253

[117] Semczuk-Sikora A Krzyzanowski A Stachowicz N : [Maternal serum concentration of angiogenic factors: PIGF, VEGF and VEGFR-1 and placental volume in pregnancies complicated by intrauterine growth restriction]. *Ginekol Pol.* 2007;78(10):783–6. 18200969

[118] Chaiworapongsa T Romero R Gotsch F : Low maternal concentrations of soluble vascular endothelial growth factor receptor-2 in pre-eclampsia and small for gestational age. *J Matern Fetal Neonatal Med.* 2008;21(1):41–52. 10.1080/14767050701831397 18175243 PMC7062305

[119] Robb KP Cotechini T Allaire C : Inflammation-induced Fetal Growth Restriction in rats is associated with increased placental HIF-1α accumulation. *PLoS One.* 2017;12(4): e0175805. 10.1371/journal.pone.0175805 28423052 PMC5397034

[120] Ashur-Fabian O Yerushalmi GM Mazaki-Tovi S : Cell free expression of hif1α and p21 in maternal peripheral blood as a marker for pre-eclampsia and fetal growth restriction. *PLoS One.* 2012;7(5): e37273. 10.1371/journal.pone.0037273 22615960 PMC3353943

[121] Pringle KG Kind KL Sferruzzi-Perri AN : Beyond oxygen: complex regulation and activity of hypoxia inducible factors in pregnancy. *Hum Reprod Update.* 2010;16(4):415–31. 10.1093/humupd/dmp046 19926662 PMC2880912

[122] Shibuya M : Vascular Endothelial Growth Factor (VEGF) and Its Receptor (VEGFR) signaling in angiogenesis: a crucial target for anti- and pro-angiogenic therapies. *Genes Cancer.* 2011;2(12):1097–105. 10.1177/1947601911423031 22866201 PMC3411125

[123] Wang Y Zhao S : Vascular biology of the placenta. In: *Colloquium Series on Integrated Systems Physiology: From Molecule to Function.*Morgan & Claypool Life Sciences,2010;2(1):1–98. 10.4199/C00016ED1V01Y201008ISP009 21452443

[124] Otrock ZK Mahfouz RA Makarem JA : Understanding the biology of angiogenesis: review of the most important molecular mechanisms. *Blood Cells Mol Dis.* 2007;39(2):212–20. 10.1016/j.bcmd.2007.04.001 17553709

[125] Kappou D Sifakis S Konstantinidou A : Role of the angiopoietin/Tie system in pregnancy (Review). *Exp Ther Med.* 2015;9(4):1091–6. 10.3892/etm.2015.2280 25780392 PMC4353758

[126] Chandrashekar VN Punnath K Dayanand KK : Malarial anemia among pregnant women in the south-western coastal city of Mangaluru in India. *Informatics Med Unlocked.* 2019;15: 100159. 10.1016/j.imu.2019.02.003

[127] Prasetyorini N Erwan NE Sardjono TW : HIF-1α regulated pathomechanism of low birth weight through angiogenesis factors in placental *Plasmodium vivax* Infection. *figshare.* Journal contribution,2021. 10.6084/m9.figshare.16577468.v4 PMC11179053 38884107

