# Supplementary material for: HIF-1α regulated pathomechanism of low birth weight through angiogenesis factors in placental Plasmodium vivax infection
Source: F1000Res. 2024 Jun 5;11:131. Originally published 2022 Feb 1. [Version 3] doi: 10.12688/f1000research.73820.3 (PMC11179053; doi:10.12688/f1000research.73820.3)
Supplement: Peer review history

# Reviewer report: Reviewer response for version 3

**DOI:** 10.5256/f1000research.167436.r286866

**Published:** 2024-06-14

**Authors:** Zainabur Rahmah

**Affiliations:** 1 Universitas Islam Negeri Maulana Malik Ibrahim Malang, Malang, Indonesia

1. The author's response is clear
2. Inclusion criteria have been listed
3. Images of placentas that did not experience placental malaria as a comparison have been added
4. For each image the type of microscope and its magnification have been completed

Is the work clearly and accurately presented and does it cite the current literature?

Yes

If applicable, is the statistical analysis and its interpretation appropriate?

Yes

Are all the source data underlying the results available to ensure full reproducibility?

Yes

Is the study design appropriate and is the work technically sound?

Yes

Are the conclusions drawn adequately supported by the results?

Yes

Are sufficient details of methods and analysis provided to allow replication by others?

Yes

Reviewer Expertise:

medical parasitology

I confirm that I have read this submission and believe that I have an appropriate level of expertise to confirm that it is of an acceptable scientific standard.

# Reviewer report: Reviewer response for version 2

**DOI:** 10.5256/f1000research.161349.r276955

**Published:** 2024-05-31

**Authors:** Zainabur Rahmah

**Affiliations:** 1 Universitas Islam Negeri Maulana Malik Ibrahim Malang, Malang, Indonesia

I enjoyed reviewing and reading Prasetyorini *et al* article about HIF-1α regulated pathomechanism of low birth weight through angiogenesis factors in placental Plasmodium vivax infection.

 Please include in the methods section how long it will take to carry out the research. The inclusion criteria for pregnant women infected with malaria have not included whether they are primigravida or multigravida, because this is related to the severity of malaria infection in the mother and fetus.

 In the results section. Is it possible to complete the image of the placental tissue in the control group as a comparison?

 In pictures 1,2 and 3. completed at the time of observation, the type of microscope used and the magnification used.

Is the work clearly and accurately presented and does it cite the current literature?

Yes

If applicable, is the statistical analysis and its interpretation appropriate?

Yes

Are all the source data underlying the results available to ensure full reproducibility?

Yes

Is the study design appropriate and is the work technically sound?

Yes

Are the conclusions drawn adequately supported by the results?

Yes

Are sufficient details of methods and analysis provided to allow replication by others?

Yes

Reviewer Expertise:

medical parasitology

I confirm that I have read this submission and believe that I have an appropriate level of expertise to confirm that it is of an acceptable scientific standard, however I have significant reservations, as outlined above.

# Response

**Published:** 2024-06-03

**Authors:** Nabila Erina Erwan

1. We will add detailed information regarding the study duration in the method section.

 2. Both primigravidae and multigravidae women were included in this study and we will add this information to the method section.

 3. We consider enriching the latest version with the normal placental tissue for comparison as suggested.

 4. All the figures will be completed with detailed information as suggested in our latest version.

# Reviewer report: Reviewer response for version 1

**DOI:** 10.5256/f1000research.77499.r182970

**Published:** 2023-08-29

**Authors:** Jean Claude Djontu

**Affiliations:** 1 Congolese Fondation for Medical Research, Brazzaville, Congo

I enjoyed reading this manuscript (Nugrahanti Prasetyorini *et al*) examining the HIF-1α regulated pathomechanism of low birth weight through angiogenesis factors in placental *Plasmodium vivax.*

 I have no major concerns with the scientific approach, but the sample size is too small (n= 19), and cannot allow to drawn high evidence based conclusion. The results of the abstract are not clearly stated, thus do not support the conclusion.

 In the method section, the authors should define the word “placental malaria” and add the the placental tissue histology as one of the tools for the placental malaria diagnosis.

 Importantly, the manuscript is in need of a linguistic revision to correct grammar, sentence structure and other language issues. I encourage the authors to engage a copyeditor, preferably with a science background.

Is the work clearly and accurately presented and does it cite the current literature?

Yes

If applicable, is the statistical analysis and its interpretation appropriate?

Yes

Are all the source data underlying the results available to ensure full reproducibility?

Yes

Is the study design appropriate and is the work technically sound?

Partly

Are the conclusions drawn adequately supported by the results?

Partly

Are sufficient details of methods and analysis provided to allow replication by others?

Yes

Reviewer Expertise:

Immunology and molecular epidemiology of malaria

I confirm that I have read this submission and believe that I have an appropriate level of expertise to confirm that it is of an acceptable scientific standard, however I have significant reservations, as outlined above.

# Response

**Published:** 2024-01-23

**Authors:** Nabila Erina Erwan

Thank you for the comments. Here are the responses for the reviewer:

- We acknowledge our limitation regarding the sample size, even though we conducted a total sampling collection for one year.
- We will increase the sample size in further research to reveal the conclusion. 
- We will clearly state the definition of placental malaria in the method section, specifically in the diagnosis of placental malaria subsection, as shown by the phrase "which is determined by placental tissue histopathological examination based on infected erythrocyte sequestration, monocyte infiltration, and hemozoin deposition (Rogerson *et al*., 2007)". 
- We will proofread our manuscript before it is resubmitted as the new version.

# Reviewer report: Reviewer response for version 1

**DOI:** 10.5256/f1000research.77499.r192254

**Published:** 2023-08-24

**Authors:** Alberto Tobón-Castaño, Carolina López-Guzman

**Affiliations:** 1 Grupo Malaria, Facultad de Medicina, Universidad de Antioquia, Antioquia, Colombia; 2 Faculty of Medicine, University of Antioquia, Medellín, Antioquia, Colombia

**General recommendations**

 Introduction:

- Organize the epidemiological information to create a stronger narrative thread for conveying the intended message. For instance, consider presenting the findings of *P. falciparum* and *P. vivax* cases by year to avoid jumping between different years or locations without a specific order.
- Upon concluding the epidemiological figures, it is worthwhile to include a conclusion, which could be positioned at the end of the second paragraph. This conclusion could emphasize that, just as *P. falciparum* has been implicated in cases of gestational malaria and low birth weight, it is important to underscore that *P. vivax* also leads to similar outcomes. Moreover, cases among pregnant women might vary in frequency based on the specific study area or endemic region being referred to.
- It is crucial to begin reinforcing the significance of *P. vivax* and its implications for infected pregnant women, as well as its association with the discovery of low birth weight, right from these initial two paragraphs.

**Inclusion Criteria: **

- Term pregnancies? The gestational weeks of the included women are not specified.
- Only reference is made to infants born with low birth weight and small for gestational age.
- Recommendation for Definitions: Similar to the definition of placental malaria, it would be beneficial to first define gestational malaria and congenital malaria. This clarification would elucidate the reason for discussing analyses of peripheral blood and cord blood samples, respectively.
- Terminology Recommendation: Clarify terms when referring to the presence of HZ in tissue; it's not about sequestration, but rather about its presence or absence. It can be found inside or outside the erythrocyte, either in a free or intraleukocytic form.
- Reference Modification: Reference 40, which relates to the count of parasitized erythrocytes and addresses parasitemia, might not be the most suitable. We suggest finding a reference related to parasitemia in humans, considering the study's context, rather than using one related to mice.

**Results Recommendation:**

- Regarding the diagnosis, all samples tested positive for *P. vivax*, and no positive samples were found for *P. falciparum*. Could a brief explanation be provided for this? Was it due to the endemic zone of the pregnant women? Did these results align with the diagnoses the pregnant women had at the time of inclusion?
- Based on the histological analysis of the placentas, was the parasitemia detected in tissue exclusively from *P. vivax*? Were the infected erythrocytes located in the intervillous space or adhered to the villi? Do you have images that can illustrate how the parasitized erythrocytes were observed in the tissue? It might be beneficial to include a panel of parasites and/or hemozoin seen in the tissue in Figure 1.
- Were the two cases of malaria diagnosed through cord blood classified as congenital malaria?

**Recommendations: Table 1:**

- It is suggested to change the order in which the data is presented, beginning with Controls and then Cases. This order aligns with how the rest of the results are shown from (Fig 2, Fig 3, Fig 4).
- It is recommended to standardize the table presentation. In Table 2, consider displaying Controls (N=14) and Cases (N=19), and remove the last row that shows the total number of cases.

**Recommendation: Figure 1:**

- For Panel A, consider selecting a more representative and clear image of an amoeboid trophozoite of *P. vivax*.
- In Panel B, the schizont appears truncated. Display a complete schizont instead.
- In Panel C, if feasible, improve the photo quality. The current image seems to have a flash glare. It should be taken without flash and with improved focus, ideally using a microscope equipped with a camera.

**Recommendation: Table 2:**

- It is recommended to change the order in which the data is presented, starting with Controls and then Cases, to maintain consistency with how the rest of the results are shown from (Fig 2, Fig 3, Fig 4).
- Bold significant P-value data. Although more of a stylistic suggestion, it helps draw the reader's attention and focuses on those key points.
- It is suggested to show the sociodemographic characteristics first, describing from the general features of the study population to the specific aspects centered around the molecular results. This approach moves from general to specific. Consider using this table as Table 1 and organizing the information based on the results section.

**Recommendation: Table 3:**

- It is recommended to change the order in which the data is presented, starting with Controls and then Cases, to maintain the consistency shown in the rest of the results from (Fig 2, Fig 3, Fig 4).
- Bold the data with significant P-values.
- Note that the P-value for the t-test and the Mann-Whitney test is significant at a level below 0.05%, only for the parameters of leukocytes and the percentile growth curve. While the results make reference to the growth curve, there is no mention of the increase in leukocytes in the cases. This should also be mentioned, as it allows for further discussion later on. This effect is expected and aligns with what has been reported for *P. falciparum* infections or previous reports on *P. vivax.*

**Recommendation: Table 4:**

- We recommend changing the order in which the data is presented, starting with Controls and then Cases, to maintain consistency with how the rest of the results are shown from (Fig 2, Fig 3, Fig 4).
- Bold the data with significant P-values.

**Subject Characteristics and Distribution: **

- We suggest placing this section first, before presenting other results related to molecular parameters such as angiogenic or hypoxia factors. It is important to describe the population characteristics of the subjects to whom various molecular parameters will be measured.

**General Recommendations for Results:**

- Ensure consistency in the presentation of decimal figures. If you are using two decimal places in tables, maintain the same format in the text and avoid using three decimal places.
- Incorporate these suggestions into the structure and formatting of your results section to enhance clarity and coherence.

**Recommendation:**

- For the section "Association of Angiogenic Factors Expression with Birth Weight": Clarify the meaning of the expression values such as VEGF (64,404±28,942), PIGF (22,814,440±9,497,663), and others like VEGFR-1 (107,444±46,696). To understand the origin of these values, specify which parameter of the ImageJ software you are using. If you are using Pixel2, explain why you chose that parameter and not the mean fluorescence intensity (IntDent) as the expression value. This question applies to other sections where expression is discussed based on an Image analysis of photos.
- The analyzes related to Low Birth Weight are not presented in detail; since all neonates had LBW, analyzes related to this outcome must be interpreted with care; it is not clear how these P values were obtained. For example: In relation to this :”VEGFR-1 expression had no direct effect on birth weight (p=0.464), but VEGFR-2 expression had a significant direct effect (p=0.020) on birth weight with an effect coefficient of 0.743”. It is not clear how this P values were obtained.
- Figure 2 and Figure 3: Since Figure 2 and Figure 3 stem from the same analysis, we recommend placing statistical analyses alongside the IFIs images. This layout would enhance clarity for the reader.
- We suggest using a scale bar in the photographs or specifying if they were captured using a mobile device or cell phone, which could explain the absence of a scale bar. This will provide clarity to the readers regarding the visual representation of the images.
- Incorporate this recommendation into your article's image captions or descriptions to enhance the understanding of the visual content.

**Summary recommendations**

**Introduction:** It's important to organize the epidemiological information coherently to effectively convey the message. It's suggested to present the findings of *P. falciparum* and *P. vivax* cases per year to avoid jumping around in time or space. After presenting the epidemiological figures, it could be concluded that, like *P. falciparum*, *P. vivax* also leads to consequences in pregnant women, influenced by the geographical zone.

**Focus on *P. vivax* and its Implications in Pregnant Women:** From the initial paragraphs, emphasize the significance of *P. vivax* in pregnant women and its relation to low birth weight.

**Inclusion Criteria:** Specify the term of gestation. Clarify that the included women had babies with low birth weight and small for gestational age. Detail the case and control groups, diagnostic methods, and sources of blood samples.

**Definitions and Terminology:** Define "gestational malaria" and "congenital malaria" first. Clarify the term "presence" of HZ in tissue. Adjust reference 40 to make it relevant to the study context.

**Results:** Explain why all samples tested positive for *P. vivax* and none for *P. falciparum*. Clarify if this is due to the endemic zone. Detail whether tissue parasitemia was exclusively from P *. vivax* and the location of infected erythrocytes in the placenta. Add clear photos of parasitized erythrocytes in tissue in Figure 1. Clarify if cases diagnosed through cord blood were classified as congenital malaria.

**Tables and Figures:** Change the presentation order in tables to start with Controls. Standardize data presentation and highlight significant P-value values. Enhance the image quality in Figure 1, specifically in panels A, B, and C. Include statistical analyses alongside IFIs images in Figure 2 and Figure 3. Use a scale bar on photographs or explain if they were taken from a mobile device.

**Presentation of Results:** Highlight the sociodemographic characteristics of subjects initially and then address the molecular results. Organize information based on the study's focus.

**General:** Maintain consistency in presenting decimal figures in tables and text. Specify ImageJ analysis parameters in sections related to angiogenic factor expression.

Is the work clearly and accurately presented and does it cite the current literature?

Partly

If applicable, is the statistical analysis and its interpretation appropriate?

Partly

Are all the source data underlying the results available to ensure full reproducibility?

No

Is the study design appropriate and is the work technically sound?

Yes

Are the conclusions drawn adequately supported by the results?

Partly

Are sufficient details of methods and analysis provided to allow replication by others?

Yes

Reviewer Expertise:

Malaria Clinical and epidemiological aspects. Malaria immunology and molecular diagnosis.

We confirm that we have read this submission and believe that we have an appropriate level of expertise to confirm that it is of an acceptable scientific standard, however we have significant reservations, as outlined above.

# Response

**Published:** 2024-01-23

**Authors:** Nabila Erina Erwan

**Introduction**

- We will reorganize the epidemiological data of malaria in our manuscript.
- As suggested, we added the conclusion of the epidemiological data at the end of the second paragraph. The conclusion will be: "This finding might emphasize that *P. vivax* also causes comparable effects, as P. falciparum has been linked to low birth weight and gestational malaria cases. Furthermore, the prevalence of instances among pregnant mothers may differ depending on the particular research location or endemic region in question."
- We mentioned the previous study in NTT, Indonesia, which showed evidence of *P. vivax*-associated low birth weight (LBW) in the second paragraph.

**Inclusion criteria**

- All the participants were in term pregnancy, and the gestational age ranged from 37 to 42 weeks (ACOG, 2013). The characteristics of the subject are shown in Table 2. We will add the definition of the term pregnancies in the characteristic of the research participant subsection.
- The inclusion criteria were pregnant women who gave birth to babies with low birth weight (LBW) and small for gestational age (SGA). The research subjects were mothers who gave birth to babies with low birth weight (< 2500 g) and small gestational age (birth weight < 10th percentile for gestational age at birth) to describe the occurrence of stunted fetal growth. 
- Our study used a peripheral blood sample to determine gestational malaria and a cord blood sample to determine placental malaria. However, we don’t identify the presence of congenital malaria. We also used placental samples to identify the placenta's pathological properties and diagnose placental malaria.
- We will change the term of hemozoin sequestration to hemozoin deposition (free hemozoin). Free hemozoin is found in extracellular form (outside the cell).
- We will change the reference with a more suitable reference for human parasitemia counting.

**Result Recommendation:**

- In Nusa Tenggara Timur, the most common species of Plasmodium is *P. vivax*, which mostly affects women (Fitri et al., 2014). The results align with the diagnoses of the pregnant women, which met the inclusion criteria.
- The infected erythrocytes are located in the intervillous space, as in Figure 2.
- The two cases, which are the infected erythrocytes found in cord blood, might not become congenital malaria because the parasite might not developed due to the presence of the IgG of the mother and the kind of fetal hemoglobin (HbF). The definitive diagnosis of congenital malaria is defined by the presence of infected erythrocytes in the peripheral blood of the fetus.

**Recommendation: Table 1:**

- We changed the order of the data presented in Table 1.
- We will edit the table presentation as suggested.

**Recommendation: Figure 1:**

- For Panel A, this is the best figure among the blood slides.
- For Panel B, most parasite morphology is not intact; it might be because the patients/subjects have antibodies against *Plasmodium*, characterized by no clinical symptoms of all the subjects (pregnant mother). This is the best figure among the blood slides.
- For Panel C, this is the best figure among the blood slides.

**Recommendation: Table 2:**

- We changed the order of the data in Table 2 and bolded the p-value data as suggested.
- We switched the data in Table 2 into Table 1 and vice versa to be presented in the text as suggested.

**Recommendation: Table 3:**

- We changed the order of the data in Table 3 and bolded the p-value data as suggested.
- We added some discussion related to the laboratory findings of the baby in the discussion section.

**Recommendation: Table 4:**

- We changed the order of the data in Table 4 and bolded the p-value data as suggested.

**Subject Characteristics and Distribution:**

- We first presented the subject characteristics and distribution in the result and discussion section, followed by molecular parameters in the study.

**General Recommendations for Results:**

- We will make sure of the consistency of decimal figures in the text.

**Recommendation:**

- The meaning of expression values of angiogenic factors expression is a semi-quantification result of the immunofluorescence image using mean fluorescence intensity (IntDent) by Image J software.
- The relationship between HIF-1a, VEGFR1, and VEGFR2 expression and birth weight was analyzed using path analysis, which describes the causal-effect relationship and the residual coefficient (e). From the model, an equation was obtained, which explained that there was no significant direct effect (p=0.99) between HIF-1∝ expression on the baby's birth weight, nor did VEGFR1 expression directly affect the baby's birth weight (p=0.46 ). However, the expression of VEGFR2 had a significant direct effect (p=0.02) on the baby's birth weight with an influence coefficient of 0.74.
- As suggested, we merged Figures 2 and 3 to place the statistical analysis alongside the IFI images.
- The figures were captured using a mobile device, which could explain the absence of a scale bar.

**Summary Recommendations:**

- We have revised the manuscript as suggested by the reviewers, and a detailed response is explained in the above response points.
